# Comparative In Vitro and In Silico Biological Evaluation of *o*-Phenylenediamine Conjugates Based on Isofunctional-Surfaced Polyanhydride and Citric Acid-Derived Carbon Quantum Nanodots

**DOI:** 10.3390/biom16101478

**Published:** 2026-10-11

**Authors:** Tutku Tunç, Gülderen Karakuş, Işık Çakmak

**Affiliations:** 1 Department of Pharmaceutical Microbiology, Faculty of Pharmacy, Sivas Cumhuriyet University, Sivas 58140, Türkiye; 2 Department of Pharmaceutical Basic Sciences, Faculty of Pharmacy, Sivas Cumhuriyet University, Sivas 58140, Türkiye; gulderen@cumhuriyet.edu.tr; 3 Vocational School of Health Services, Karadeniz Technical University, Trabzon 61080, Türkiye; isikcakmak@ktu.edu.tr

**Keywords:** anhydride surface modification, *o*-phenylenediamine, maleic anhydride-vinyl acetate copolymer, citric acid-based carbon quantum nanodots, anticancer-antimicrobial activity, molecular docking interactions

## Abstract

This study aimed to comparatively evaluate the anticancer and antimicrobial activities of conjugates formed from poly(maleic anhydride-co-vinyl acetate) (MAVA) and carbon quantum dots (CNDs) with *o*-phenylenediamine (*o*-PDA). *o*-PDA was chosen as the conjugation agent due to its status as a significant aromatic six-membered diamine, which enables specific reactions in the field of drug discovery. MAVA and CNDs, which are low-toxicity carrier systems, were prepared according to a previously developed protocol and conjugated with *o*-PDA to yield MAVA/*o*-PDA and CNDs/*o*-PDA. The structural characteristics of the conjugates were confirmed by ATR-FTIR and ^1^H-NMR analyses. The cytotoxic effects of the obtained conjugates on MG-63, A549, and MCF-7 cancer cell lines and healthy WI-38 cells, as well as their antimicrobial activities against various Gram-positive and Gram-negative bacteria, were evaluated. Additionally, ADMET properties and drug-likeness profiles were analyzed in silico to support the biological findings. While neither conjugate induced significant cytotoxicity in healthy WI-38 cells, they exhibited a stronger cytotoxic effect in cancer cells compared to *o*-PDA alone and the carrier systems. CNDs/*o*-PDA was found to be more effective than MAVA/*o*-PDA in MG-63, A549, and MCF-7 cells, whereas MAVA/*o*-PDA exhibited stronger activity in C6 cells. The conjugates also exhibited higher antimicrobial activity than their individual components, particularly against Gram-negative bacteria. In general, it has been observed that conjugation with *o*-PDA can enhance the biological activities of MAVA and CNDs while limiting toxicity in normal cells.

## 1. Introduction

The developing field of nanomedicine offers innovative strategies for the diagnosis and treatment of diseases by combining the precision of molecular targeting with the versatility of therapeutic delivery systems. Polymer–drug conjugates (PDCs), a key approach in this field, demonstrate significant potential for controlled, targeted drug delivery by combining the efficacy of therapeutic agents with the customizable properties of polymers [1]. In particular, the development of biocompatible and biodegradable polymers that enable integration with biological systems has marked a significant turning point in drug delivery, tissue engineering, and the development of medical devices [2]. Pharmacologically active polymers offer advantages in drug discovery and therapeutic applications by providing properties not easily achieved with traditional small-molecule drugs. The role of polymers in therapeutic systems is not limited to that of a passive carrier; some polymer structures can exhibit direct biological activity. In this case, the polymer component serves as a structural framework that determines the physicochemical properties of the conjugate, while also contributing to therapeutic efficacy through biological interactions [3,4].

One of the most significant advantages is the ability to modify the physicochemical properties of the material for specific applications by changing the type of comonomer and the polymer architecture. This enables various drugs and biologically active molecules to be covalently attached to the polymer chain through the reaction of the anhydride rings present in maleic anhydride copolymers with nucleophilic groups. This allows the development of attractive polymer–drug conjugates with properties such as hydrophilici-ty/hydrophobicity, water solubility, and surface characteristics that can be tuned to meet application requirements thanks to the reactivity of maleic anhydride [5,6].

In recent years, there has been a growing recognition of the importance of antimicrobial polymers, particularly in the context of increasing resistance to microbes. These polymers are being considered as potential alternatives to traditional antimicrobial agents [7,8]. It is important to note that polymers that demonstrate antimicrobial properties can either exhibit their own antimicrobial activity directly or be utilized in combination with, or in conjunction with, other antimicrobial compounds, such as antibiotics [9]. Maleic anhydride-based polymers are cited as examples of polymeric materials that exhibit antimicrobial properties (and antitumor activity), and are being evaluated in various industrial and biomedical applications [10,11]. As demonstrated by extant literature, poly(maleic anhydride-*co*-vinyl acetate) (MAVA) copolymers and polymer–drug conjugates developed based on this copolymer have been shown to exhibit cytotoxic, antimicrobial, and anticancer activities in different cancer cell models [3,12]. Conversely, carbon dots (CDs) have garnered significant attention due to their advantageous optical properties, cost-effectiveness, low toxicity, and biocompatibility, positioning them as a prominent subject in biomedical research [13]. Furthermore, CNDs are frequently favored over toxic heavy-metal quantum dots on account of their favorable properties, including chemical inertness, high water solubility, and chemical stability. These properties render them biologically ideal, eco-friendly, and harmless materials, as well as potential nanocarriers for drug delivery and biomedical applications [14,15]. However, recent studies have demonstrated that, depending on their synthesis sources, carbon dots and polymers can be regarded not only as carriers but also as functional materials with the capacity to exhibit direct biological or therapeutic activity [13].

The phenylenediamine (PDA) molecule is a disubstituted benzene derivative with three isomers: *o*-, *m*-, and *p*-phenylenediamine. It is an aromatic six-membered ring structure also classified as an aniline derivative. This molecule has enabled the development of various heterocyclic compounds, such as fungicides, corrosion inhibitors, and pharmaceuticals. *o*-PDA is an aromatic compound formed by the attachment of two amino groups to a benzene ring and exists as *o*-, *m*-, and *p*-isomers [16]. In particular, *o*-PDA is an important starting material used in the synthesis of various heterocyclic structures, and its derivatives have been studied for their diverse biological activities [17,18]. In this study, *o*-PDA was selected as a component used in the functionalization of MAVA and CNDs surfaces; accordingly, MAVA/*o*-PDA and CNDs/*o*-PDA derivatives were prepared.

This study focuses on developing a practical method for the conjugation of small molecules containing amino groups. This innovative approach sheds light on the fundamental structural developments of materials with different synthetic backgrounds and common surface properties. The primary objective of the study is to develop a suitable method for determining the relative binding efficiency and yield of the *o*-PDA conjugation agent with linear and spherical surfaces. A subsequent objective is to investigate how the resulting conjugates interact with targeted structures, such as cancer and bacterial cells, depending on their surface properties and shapes. Surface functionalization plays a crucial role in the development of biomedical molecules because it enables the imparting of desired properties while eliminating issues such as biocompatibility, toxicity, and solubility. The selected linear maleic anhydride (MAVA) copolymer and spherical quantum nanodots (CNDs) share a common structural feature: the R-C(=O)-O-C(=O)-R functionality, which is regularly distributed on the anhydride rings on their surfaces. This makes these structures suitable for conjugation with small drug molecules bearing primary amine (-NH_2_) groups and their derivatives. Conjugation can be easily achieved under mild conditions via a nucleophilic ring-opening reaction, thereby yielding polymer–drug or quantum dot–drug complexes free of unwanted side effects or bioavailability issues.

Determining the biological potential of the conjugates obtained requires not only identifying their cellular and microbiological effects but also evaluating the possible mechanisms of these effects at the molecular level. Therefore, supporting experimental biological findings in the context of potential interactions with target molecules can contribute to a more comprehensive understanding of the structure–activity relationships of the conjugates. In recent years, bioinformatics and other computational approaches have been increasingly used, particularly in cancer research, to identify potential therapeutic targets and drug candidates [19]. Molecular docking, one of these approaches, can provide information about the potential mechanisms of action of drug candidates by predicting ligand-receptor interactions and evaluating potential binding sites [20,21].

In this context, investigating the molecular interactions that may underlie experimentally observed cytotoxic effects requires the selection of an appropriate biological target. In this study, the Epidermal Growth Factor Receptor (EGFR) was selected as a primary molecular target to establish a rational link between computational molecular docking simulations and observed in vitro cytotoxicity results. EGFR is a transmembrane tyrosine kinase that is overexpressed in a wide range of solid tumors, including lung (A549), breast (MCF-7), bone (MG-63), and glial (C6) malignancies, and regulates cell proliferation and survival. Gene expression data from The Human Protein Atlas (HPA) confirmed high EGFR levels in the selected cell lines and provided a valid biological basis for our anticancer evaluation.

## 2. Materials and Methods

### 2.1. Synthesis and Characterization of Materials

All reagents and chemicals used in the synthesis, passivation, and purification processes are of analytical grade. The carrier materials that are the subject of this study—namely, maleic anhydride-vinyl acetate copolymer (MAVA) and citric acid-based carbon quantum dots (CA-based CNDs)—were prepared using charge-transfer complex polymerization and stepwise thermal pyrolysis methods, respectively. The conjugated forms of both carriers were obtained by surface functionalization with *o*-PDA. This synthesis route and functionalization procedure are based on our previous work [22,23].

#### 2.1.1. Synthesis of Carrier-I (MAVA)

MAVA copolymer was conducted in accordance with the conventional method. The MAVA copolymer was synthesised according to radical chain polymerisation using the in situ charge transfer complex (CTC). In summary, the substance was synthesised by free-radical copolymerisation from the starting monomers, maleic anhydride (MA, Merck Schuchardt, Germany) and vinyl acetate (VA, Sigma-Aldrich, St. Louis, MO, USA)), with a monomer feeding mole ratio of 1:1 (MA:VA) in methyl ethyl ketone (MEK, Merck, Schuchardt, Germany). The process was initiated with a powerful benzoyl peroxide (BPO, Merck, Schuchardt, Germany)-free radical initiator and was carried out at approximately 80 °C for 24 h. The resulting polymeric powder was then precipitated from the organic solvent-based solution, washed, and purified by drying in a vacuum oven at 50 °C for 24 h [12].

#### 2.1.2. Synthesis of Carrier-II (CNDs)

The synthesis of citric acid (CA, Merck, Schuchardt, Germany)-based carbon quantum nanodots was optimised through thermal pyrolysis, which involved the gradual thermal decomposition of CA [24]. In summary, anhydrous CA was introduced into an open round-bottom flask and subsequently heated at a constant rate to 180 °C within a reaction system that was equipped with Radleys. This process was conducted without interruption to the heating sequence. The colour of the liquid in the reaction medium underwent a change from colourless to pale yellow at the onset of pyrolysis. Within half an hour, the colour transitioned to orange as the first CNDs began to form. With the continuation of the heating process, the orange liquid rapidly turned brown within one hour. The general procedure for synthesising the acid anhydride-CNDs involves heating the anhydrous CA at 180 °C for two hours, resulting in the formation of a brown powder residue known as the nanodots. The synthesis of CNDs was conducted with the specific intention of employing them for surface modification, without the necessity of additional purification [22].

#### 2.1.3. The Process of *o*-PDA Surface Functionalization of Carrier-I and Carrier-II

In the present study, both MAVA copolymer and CA-based CNDs in conjugated forms (MAVA/*o*-PDA and CNDs/*o*-PDA) were prepared by modifying MAVA and CNDs with *o*-PDA(Merck, Schuchardt, Germany) conjugating agent in a molar ratio of 1:1 in anhydrous dimethylformamide (DMF, Merck, Schuchardt, Germany) as organic solvent, using triethylamine (TEA, Carlo-Erba, Rodano, Italy) as a conjugation catalyst. The modified conjugates were prepared over a period of 6 h/16 h at 40 °C. The freeze-dried powder of MAVA and CNDs was subjected to gentle magnetic stirring until the powders dissolved in well-stirred DMF, thereby yielding a homogeneous solution. The initial step in the conjugation reaction was initiated by the addition of a minute quantity of the catalyst, TEA, to the well-stirred solutions of MAVA-DMF and CNDs-DMF. Subsequently, the mutual agent *o*-PDA molecule was dissolved in anhydrous DMF and mixed rapidly by dropwise addition into the previously prepared MAVA-DMF and CNDs-DMF mixed solutions at room temperature. The final mixed solutions were provided with a reflux condenser according to the Radley system and thoroughly stirred in the dark at 40 °C for 6 h (MAVA conjugation) and 16 h (CNDs conjugation) by shaking at 400 rpm until a viscous solution was formed. The reaction was then halted and allowed to cool to room temperature. The reaction mediums were then subjected to vacuum filtration and repeatedly purified with an excess of cold diethyl ether. The final precipitates were also purified by centrifugation at 6000 rpm for 5 min, yielding a yellow-orange powder. This was then incubated in a vacuum incubator at 50 °C for 24 h, resulting in the formation of the MAVA/*o*-PDA and CNDs/*o*-PDA conjugates [22,23].

#### 2.1.4. Structural Characterization

The chemical structures and conjugation mechanisms of all synthesized samples were characterized in detail in our previous studies using attenuated total reflection Fourier transform infrared spectroscopy (ATR-FTIR), nuclear magnetic resonance spectroscopy (^1^H-NMR), fluorescence spectroscopy, and UV-visible spectroscopy. Additionally, the crystal structure of the CNDs samples was investigated using X-ray diffraction (XRD). Thermogravimetric analysis (TGA) and differential scanning calorimetry (DSC) were performed for physical characterization; liquid chromatography–quadrupole time-of-flight mass spectrometry (LC-QTOF-MS) and size-exclusion chromatography (SEC) were used for molecular weight and purity analyses [22,23]. Detailed structural characterization data for all synthesized samples (MAVA/MAVA-*o*-PDA and CNDs/CNDs-*o*-PDA) have been provided in the Supplementary File.

### 2.2. EGFR Expression Analysis/EGFR Expression in Cancer Cell Lines

The Human Protein Atlas (HPA) provides a comprehensive data resource on protein expression, along with immunohistochemically stained images of tissue samples. Within the HPA, protein expression is classified based on organs, tissues, cell types, and disease pathologies, enabling researchers to examine the distribution and expression profiles of specific proteins in the human body. The data provided by the HPA are openly accessible and allow for the evaluation of the expression of individual genes and gene groups in different human tissues and cell types [25].

In this study, the expression of the Epidermal Growth Factor Receptor (EGFR) in cancer cell lines was examined using the “Cell Line” database of The Human Protein Atlas. Cell line-based expression data for EGFR were obtained from the HPA on September 1, 2026. Studies systematically evaluating gene expression profiles in cell lines demonstrate that HPA data can be used to investigate the molecular characteristics of different cancer cell models [26].

### 2.3. Anticancer Activity

Human lung cancer cells (A549, ATCC CCL-185), human breast cancer cells (MCF-7, ATCC HTB-22), rat glioma cells (C6, ATCC CCL-107), human osteosarcoma cells (MG-63, ATCC CRL-1427), and normal human lung fibroblast cells (WI-38, ATCC CCL-75) were obtained from ATCC (American Type Culture Collection, USA) as part of our previously conducted projects. The cytotoxic effects of the synthesized compounds on these cell lines were evaluated using the XTT (2,3-bis(2-methoxy-4-nitro-5-sulfophenyl)-2H-tetrazolium-5-carboxanilide) assay.

First, the cells were passaged and cultured until they reached the appropriate density for the specific experiment. Then, 10^4^ cells were seeded into each microplate well. Six different concentrations (3.125, 6.25, 12.5, 25, 50, and 100 µg/mL) of CNDs, *o*-PDA, MAVA, CNDs/*o*-PDA, and MAVA/*o*-PDA were applied to the cells. Doxorubicin hydrochloride (D1515; Sigma-Aldrich, St. Louis, MO, USA) was used as the positive control, while dimethyl sulfoxide (DMSO) was used as the negative control. The microplates were incubated at 37 °C in a humidified atmosphere containing 5% CO_2_ for 24 h [27].

At the end of the incubation period, the culture medium (DMEM supplemented with FBS and penicillin-streptomycin) was aspirated from each well. Finally, 100 µL of XTT solution, prepared according to the experimental protocol, was added to each well. The microplates were then incubated for 4 h, and optical density was measured at 495 nm using a Spectrostar Nano microplate reader (BMG LABTECH, Ortenberg, Germany). All experiments were conducted in triplicate, and the results are expressed as the mean ± standard deviation (SD).

The selectivity index, representing the ratio of the IC_50_ values of the synthesized compounds in normal cells to those in cancer cells, was calculated using the relevant formula and showed that this compound affected cancer cells much more specifically than normal cells: fibroblast IC_50_/cancer cell IC_50_ [28].

### 2.4. Antimicrobial Activity

The Minimum Inhibitory Concentration (MIC) test was used to determine the antimicrobial activity of CNDs, *o*-PDA, CNDs/*o*-PDA, MAVA, and MAVA/*o*-PDA against pathogenic microorganisms. The pathogenic microorganisms used in the experiment (*Staphylococcus aureus*-ATCC 29213, *Enterococcus faecalis*-ATCC 29212, *Streptococcus mutans*-ATCC 25175, *Bacillus cereus*-ATCC 11778, *Pseudomonas aeruginosa*-ATCC 27853, *Escherichia coli*-ATCC 25922, *Klebsiella pneumoniae*-ATCC 13883, *Candida albicans*-ATCC 10231) were cultured on blood agar, Mueller–Hinton agar (HiMedia, Maharashtra, India), and Sabouraud dextrose agar (Scharlau, Barcelona, Spain). The concentration of all microorganisms in Mueller–Hinton broth (HiMedia, Maharashtra, India) was adjusted to 0.5 McFarland. For the MIC test, the compounds were dissolved in DMSO and added to a U-shaped plate in 10 concentrations. Microorganisms prepared according to the McFarland standard were then added to the plates. After preparing the reference antibiotics, growth control wells, and sterilization control wells, the entire plate was incubated at 37 °C for 24 h. The first well in which no growth was observed after incubation was determined to be the MIC value. All tests were performed in triplicate [29,30].

### 2.5. In Silico Analysis

#### 2.5.1. Molecular Docking

The crystal structure of the enzyme target used in this study was obtained from the RCSB Protein Data Bank (PDB) [31]; there are four anticancer targets and eight antimicrobial targets. Epidermal Growth Factor Receptor (EGFR) (PDB ID:1XKK) for anticancer study, *Staphylococcus aureus*: DNA gyrase (GyrB) (PDB ID:6TTG), Enterococcus faecalis: Dihydrofolate reductase (DHFR) (PDB ID:4M7V), *Streptococcus mutans*: Sortase A (PDB ID:4TQX), *Bacillus cereus*: Metallo-beta-lactamase type 2 (PDB ID:6F2N), *Pseudomonas aeruginosa*: LasR (quorum sensing (PDB ID:4JVI), *Escherichia coli*: DNA gyrase B (PDB ID:7P2N), *Klebsiella pneumoniae*: SHV-1 beta-lactamase (PDB ID:2ZD8), *Candida albicans*: Lanosterol 14α-demethylase (CYP51) PDB ID:5TZ1) for antimicrobial study. Prior to protein docking analysis, it was prepared using the Chimera 1.16 [32] program and saved in .pdb format. During preparation, ions, water, and ligand structures within the protein were cleaned, and H and Gasteiger charges were added. The prepared protein was saved in .pdbqt format using AutoDockTools-1.5.6 [33]. The compound structure used for docking was saved in sdf format. The ligand structure was optimized using Open Babel 3.1.1 [34] and converted to pdbqt format. Hydrogen atoms were added to ligands lacking hydrogen atoms using Avogadro 1.2.0 [35].

A grid box was defined to determine the binding region for docking studies. For the anticancer targets, EGFR (PDB ID: 1XKK) grid box was x: 12.850, y: 31.868, z: 36.357 with box size 30 × 30 × 30 Å. For the antimicrobial targets, the *Staphylococcus aureus* GyrB (PDB ID: 6TTG) grid box was x: 3.889, y: 2.392, z: −0.426 with box size 24 × 24 × 24 Å. *Enterococcus faecalis* DHFR (PDB ID: 4M7V) grid box was x: 26.353, y: −13.255, z: 11.852 with box size 24 × 24 × 24 Å. *Streptococcus mutans* Sortase A (PDB ID: 4TQX) grid box was x: 6.331, y: 26.758, z: −11.095 with box size 28 × 28 × 28 Å. *Bacillus cereus* metallo-β-lactamase type 2 (PDB ID: 6F2N) grid box was x: 12.915, y: 13.279, z: 43.455 with box size 22 × 22 × 22 Å. *Pseudomonas aeruginosa* LasR (PDB ID: 4JVI) grid box was x: −33.378, y: 54.745, z: 11.417 with box size 26 × 24 × 28 Å. *Escherichia coli* DNA gyrase B (PDB ID: 7P2N) grid box was x: −19.226, y: −15.146, z: 7.124 with box size 26 × 26 × 26 Å. *Klebsiella pneumoniae* SHV-1 β-lactamase (PDB ID: 2ZD8) grid box was x: 9.506, y: 34.995, z: 2.655 with box size 28 × 28 × 28 Å. *Candida albicans* CYP51 (PDB ID: 5TZ1) grid box was x: 70.674, y: 65.297, z: 4.494 with box size 28 × 28 × 28 Å. A grid box with a 1.0 Å spacing was defined to determine the binding region for docking studies. The co-crystallized ligand, which forms a complex with the target protein, was used as the reference ligand in this study. Molecular docking analysis was performed using AutoDock Vina 1.2.3 [36]. Chimera 1.16 [32], Discovery Studio Visualizer 2025 [37], and PyMOL (Version 3.1.3.1) [38] were used to visualize and analyze the docking results.

#### 2.5.2. ADMET and Drug-Likeness Analysis

Although a large number of biologically active compounds have been identified using computational methods, the conversion rate to drug candidates remains low due to shortcomings in ADMET (Absorption, Distribution, Metabolism, and Excretion) properties and toxicity issues [39]. As in vivo and in vitro studies are limited in terms of both time and cost, the use of in silico methods has come to the fore in evaluations. Today, many analyses, including the prediction of ADMET properties, can be carried out effectively with the aid of various computational in silico tools [40]. The physicochemical and pharmacokinetic properties of the molecules were evaluated in silico using SwissADME (http://www.swissadme.ch, accessed on 1 September 2026) [41]. Bioavailability potential was determined by examining physicochemical parameters such as lipophilicity, size, polarity, solubility, flexibility, and saturation in radar plots. Gastrointestinal absorption and blood–brain barrier penetration were predicted using the boiled-egg model. These graphs facilitate the candidate selection process by enabling a rapid and visual assessment of the compounds’ pharmacokinetic properties, thereby facilitating the more efficient and rapid identification of molecules with drug-like properties. Lipinski’s Five Rules [42] were used as a filter for drug similarity. Predicting the toxicity potential of compounds constitutes a key stage in the drug design and development process. Computational toxicity prediction methods not only provide results more quickly than experimental studies but also contribute to the reduction in animal testing. In this context, the ProTox 3.0 online toxicity prediction tool was used to evaluate the toxicity profiles of the compounds [43].

### 2.6. Statistical Analysis

The normality of the data was assessed using the Shapiro–Wilk test. Comparisons between two groups were performed using the independent samples t-test for normally distributed data or the Mann–Whitney U test for non-normally distributed data. Comparisons among three or more groups were performed using one-way ANOVA, followed by Tukey’s HSD post hoc test, or the Kruskal–Wallis test, followed by Dunn’s post hoc test, as appropriate. Data are presented as mean ± standard deviation (mean ± SD). Statistical analyses were performed using IBM SPSS Statistics (version 22.0; IBM Corp., Armonk, NY, USA), and a *p*-value of <0.05 was considered statistically significant.

## 3. Results

**Scheme 1:**
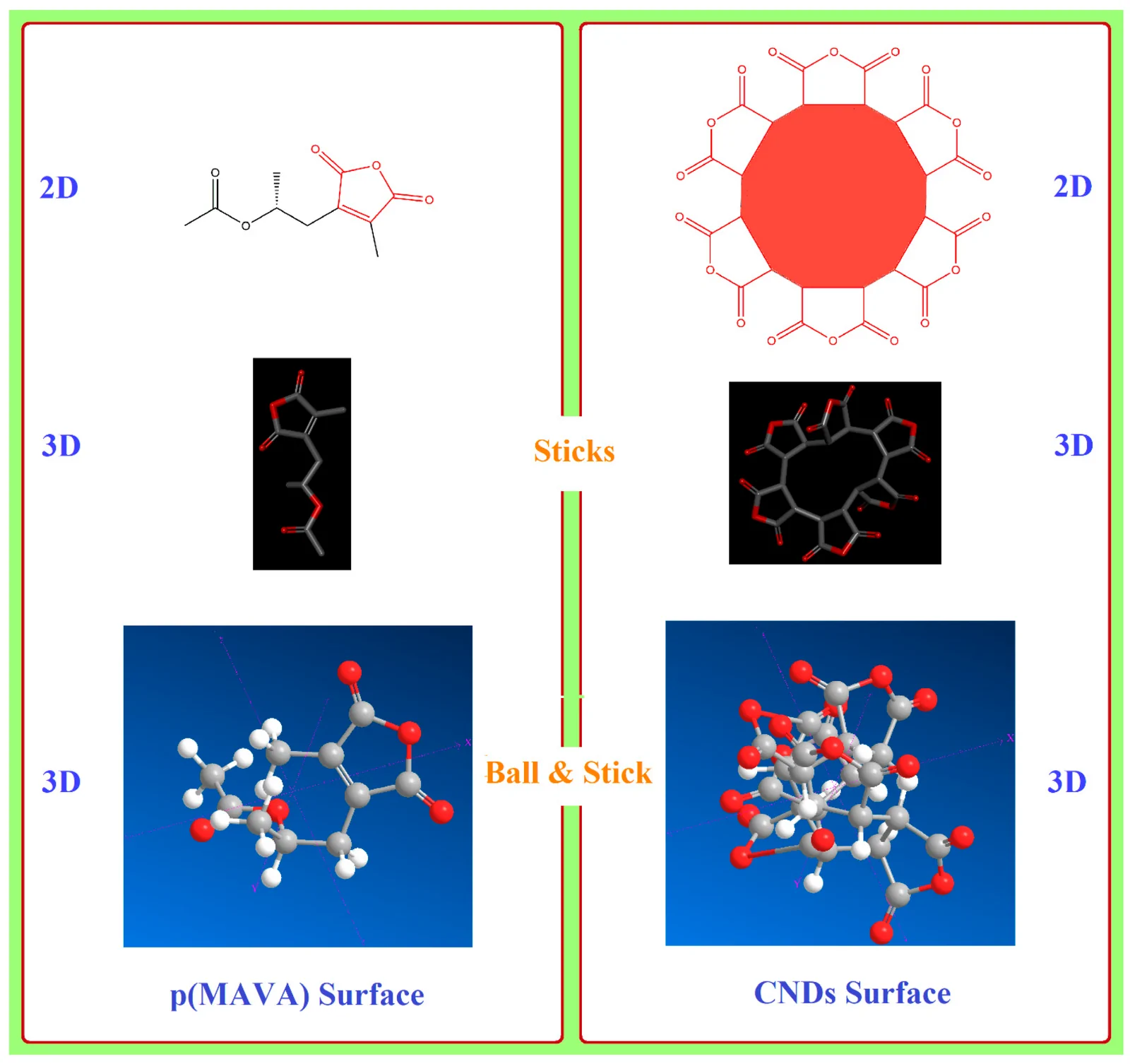
Schematic illustration of the homogeneous distribution of functional anhydride rings on the surface of a linear polyanhydride-based copolymer and on the surface of spherical quantum nanodots. In the black figure, red represents carbonyl groups, while gray represents the atoms in the remaining chain structure. In the blue figures, red, gray, and white represent oxygen (O), carbon (C), and hydrogen (H) atoms, respectively.

In this study, conjugates formed using poly(maleic anhydride-*co*-vinyl acetate) (MAVA) and carbon quantum dots (CNDs) with *o*-phenylenediamine (*o*-PDA) were used. As visualized in Scheme 1, the functional anhydride ring on the surface of the selected polyanhydride-based copolymer and carbon quantum nanodots is similar. The special structures of both CNDs and MAVA make them particularly suitable for modification, especially in terms of the functionality of their external surfaces. The uniformly distributed anhydride ring units facilitate the formation of amides accompanied by carboxylic acids—a regular bi-functional structural transformation by triggering ring-opening reactions with amine-containing compounds. The flat and spherical equivalent surfaces of these two functional structures allow nucleophilic ring-opening to form suitable conjugates with drugs or pharmaceutically active substances.

**Scheme 2:**
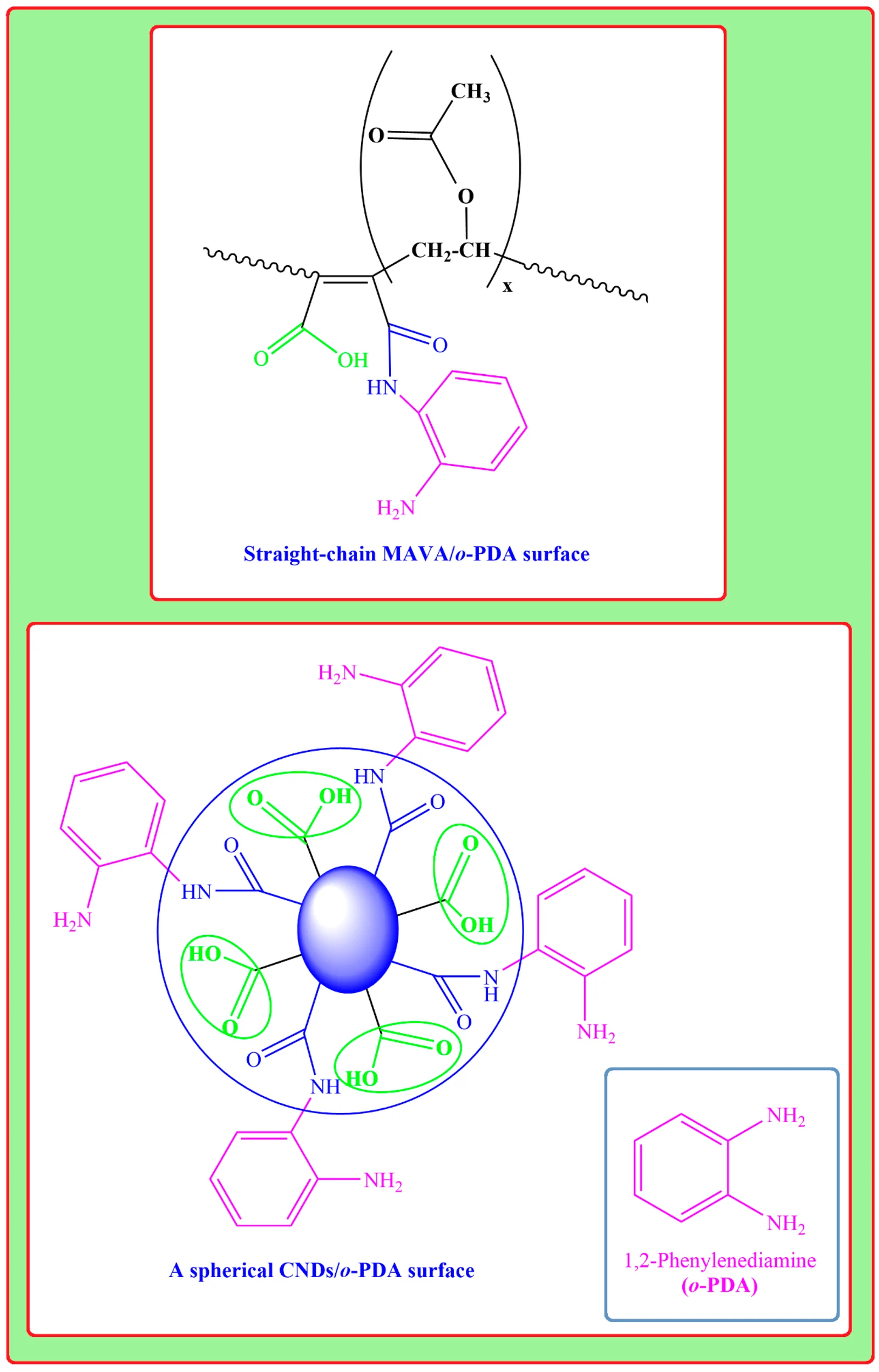
Schematic representation of homogeneous distribution, showing *o*-PDA conjugated to the surface of a linear polyanhydride-based copolymer and spherical quantum nanodots, following a ring-opening reaction.

As demonstrated in our previous publication, spectroscopic analysis has confirmed the water solubility of MAVA- and CNDs-based conjugates, thus validating the efficacy of the conjugation process following the ring-opening reaction, which occurs via an amide mechanism. The process, in which acid anhydrides on MAVA and CNDs react with amines (represented by *o*-PDA) to form amides and carboxylic acid functional groups, can be summarized by the following single-step mechanism: The sequence of events begins with a nucleophilic attack by the primary aromatic amine molecule on the anhydride group. This is followed by a ring-opening reaction, deprotonation performed by the aromatic amine following the nucleophilic attack, and finally, proton transfer or intramolecular bonding (Scheme 2).

### 3.1. Expression Profile of EGFR in Cancer Cell Lines

The epidermal growth factor receptor (EGFR) is a cell surface receptor that plays a role in regulating processes such as cell growth, proliferation, and differentiation in normal cells. However, overexpression, activation, or mutations of EGFR have been linked to tumor development and progression in many types of cancer. In this study, EGFR expression profiles in various cancer types and cell lines—including those evaluated for anticancer activity—were examined using data from The Human Protein Atlas (HPA), and the resulting data are presented in Figure S1 in the Supplementary File.

### 3.2. Anticancer Activity Results

The IC_50_ values determined from cytotoxicity assays (XTT assay) conducted on four different cancer cell lines (A549, MG-63, MCF-7, and C6) and a healthy cell line (WI-38) are shown in Table 1.

The cytotoxic activities of CNDs, *o*-PDA, CNDs/*o*-PDA, MAVA, and MAVA/*o*-PDA samples on C6, MCF-7, MG-63, and A-549 cancer cells, as well as WI-38 normal fibroblast cells, were evaluated using IC_50_ values (Table 1). The IC_50_ values of the CNDs, *o*-PDA, CNDs/*o*-PDA, MAVA, and MAVA/*o*-PDA in WI-38 normal fibroblast cells were >100 µg/mL, suggesting that these test materials exhibit low cytotoxic effects on normal cells and may possess selective anticancer activity against cancer cells.

CNDs/*o*-PDA exhibited lower IC_50_ values compared to CNDs and *o*-PDA in all cancer cell lines, and these differences were found to be statistically significant (*p*^1^ < 0.0001; *p*^2^ < 0.00001). Similarly, MAVA/*o*-PDA exhibited higher cytotoxic activity compared to MAVA and *o*-PDA (*p*^1^ < 0.0001; *p*^3^ < 0.00001). The lowest IC_50_ value for MAVA/*o*-PDA was observed in C6 cells (6.50 ± 0.01 µg/mL), and for CNDs/*o*-PDA, it was observed in MG-63 cells (9.42 ± 0.54 µg/mL).

In the comparison between CNDs and MAVA, MAVA exhibited a higher cytotoxic effect in C6 (*p*^4^ = 0.0009) and MG-63 (*p*^4^ = 0.005) cells, whereas no significant difference was observed in MCF-7 (*p*^4^ = 0.90) and A549 (*p*^4^ = 0.34) cells. When CNDs/*o*-PDA was compared with MAVA/*o*-PDA, MAVA/*o*-PDA was found to be significantly more potent only in C6 cells (*p*^5^ < 0.0001). In contrast, no statistically significant difference was found between the two test materials in MCF-7 (*p*^5^ = 0.12) and MG-63 (*p*^5^ = 0.08) cells, while in A549 cells, CNDs/*o*-PDA was found to have a lower IC_50_ value compared to MAVA/*o*-PDA (*p*^5^ = 0.00003).

When the CNDs, *o*-PDA, and CNDs/*o*-PDA triad are evaluated individually, it is observed that the IC_50_ values of the newly synthesized CNDs/*o*-PDA are significantly more effective than those of its components across all cancer cell lines (C6 = 11.47, MCF-7 = 13.28, MG-63 = 9.42, A549 = 12.06). Since low IC_50_ values are associated with high cytotoxic potential, it can be concluded that CNDs/*o*-PDA exhibits a highly effective cytotoxic potential against MG-63 (osteosarcoma) cells (IC_50_ = 9.42). When the trio of MAVA, *o*-PDA, and MAVA/*o*-PDA is evaluated on its own, it is observed that the IC_50_ values, particularly for the C6 and MCF-7 cell lines, are significantly lower than those of the individual components (C6 = 6.50, MCF-7 = 14.81). Among all tested materials, MAVA/*o*-PDA stands out as the formulation with the highest cytotoxic activity against C6 cells, with an IC_50_ of 6.50.

Overall, the findings demonstrate that formulations combining CNDs and MAVA with *o*-PDA exhibit statistically significantly higher anticancer activity (*p* < 0.05) compared to the application of CNDs and MAVA alone, and that they possess selective cytotoxic activity while exhibiting low toxicity in normal cells.

**Figure 1:**
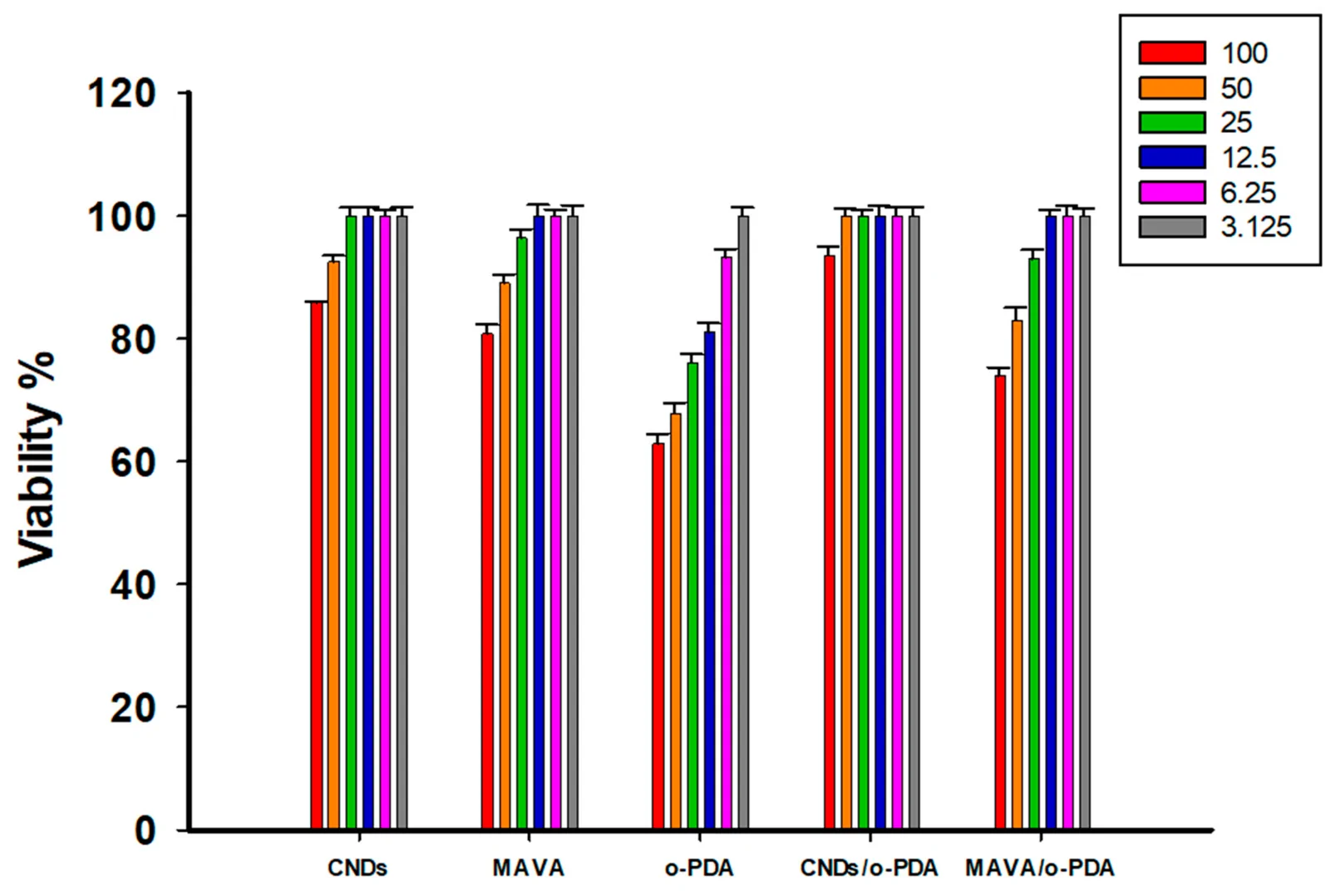
Cell viability of CNDs, *o*-PDA, CNDs/*o*-PDA, MAVA, and MAVA/*o*-PDA on WI-38 cells.

The results of the cytotoxicity tests of the synthetic compounds and their components on WI-38 cells are shown in Figure 1. It was found that CNDs/*o*-PDA and MAVA/*o*-PDA exhibited almost no toxic effects when compared to their components.

The cytotoxic effects of all compounds on WI-38 cells were examined in the concentration range of 3.125–100 µg/mL. Although a gradual decrease in cell viability was observed at increasing concentrations, since none of the tested doses reduced viability below the 50% threshold, the IC_50_ value could not be calculated directly and was therefore assumed to be a value exceeding 100 µg/mL. In conclusion, these findings indicate that none of the compounds produced significant cytotoxic effects on WI-38 cells within the concentration range examined.

The selectivity index values for CNDs, *o*-PDA, CNDs/*o*-PDA, MAVA and MAVA/*o*-PDA are presented in Table 2.

Based on the results of the anticancer activity tests, it was determined that both synthesized compounds were highly effective against all cancer cell lines tested and exhibited high selectivity along with low IC_50_ values. The selectivity index (SI) is a parameter that indicates a compound’s selective toxicity toward cancer cells compared to normal cells; it is calculated by dividing the IC_50_ value in healthy cells by the IC_50_ value in cancer cells (SI = IC_50_[WI-38]/IC_50_[cancer cell]). A high SI value indicates that the compound exhibits a more selective cytotoxic effect against cancer cells compared to normal cells.

Upon examining the SI values presented in Table 2, it is observed that CNDs/*o*-PDA exhibits significantly higher SI values across all cancer cell lines (C6 = 8.72, MCF-7 = 7.53, MG-63 = 10.62, A549 = 8.29) compared to both CNDs and *o*-PDA alone. The SI values for doxorubicin, used as a positive control, were determined to be 5.24, 3.69, 4.06, and 7.51 in the C6, MCF-7, MG-63, and A549 cells, respectively; CNDs/*o*-PDA demonstrated higher selectivity than doxorubicin in all cancer cell lines. The highest SI value for CNDs/*o*-PDA was obtained in MG-63 cells (10.62), a finding that supports the formulation’s highly selective effect against osteosarcoma cells. CNDs and *o*-PDA, on the other hand, exhibited SI values that were higher than those of doxorubicin in MCF-7 and MG-63 cells, similar to or slightly lower than those of doxorubicin in C6 cells, and lower than those of doxorubicin in A549 cells.

When the trio of MAVA, *o*-PDA, and MAVA/*o*-PDA was evaluated individually, it was determined that MAVA/*o*-PDA achieved the highest selectivity index among all samples, particularly in C6 cells (SI = 15.38). This value is approximately three times the SI value of doxorubicin (5.24) in the same cell line. This finding is also consistent with the IC_50_ results, in which MAVA/*o*-PDA exhibited the lowest IC_50_ value (6.50 µg/mL) in C6 cells, demonstrating that MAVA/*o*-PDA exerts a cytotoxic effect against C6 cells that is both potent and highly selective. In contrast, the SI values of MAVA/*o*-PDA in MCF-7 (6.75), MG-63 (6.67), and A549 (6.55) cells were similar to one another and remained at a more moderate level. These values were higher than the SI values for doxorubicin in MCF-7 and MG-63 cells (3.69 and 4.06, respectively) but lower than the SI value for doxorubicin in A549 cells (7.51). Similarly, MAVA exhibited higher selectivity than doxorubicin in C6 (9.19), MCF-7 (5.02), and MG-63 (5.09) cells, but lower selectivity in A549 cells (6.19).

In general, the SI values support the finding that formulations combined with *o*-PDA (particularly MAVA/*o*-PDA and CNDs/*o*-PDA) offer a therapeutic index that is mostly broader between cancerous and normal cells than that provided by their individual components.

**Figure 2:**
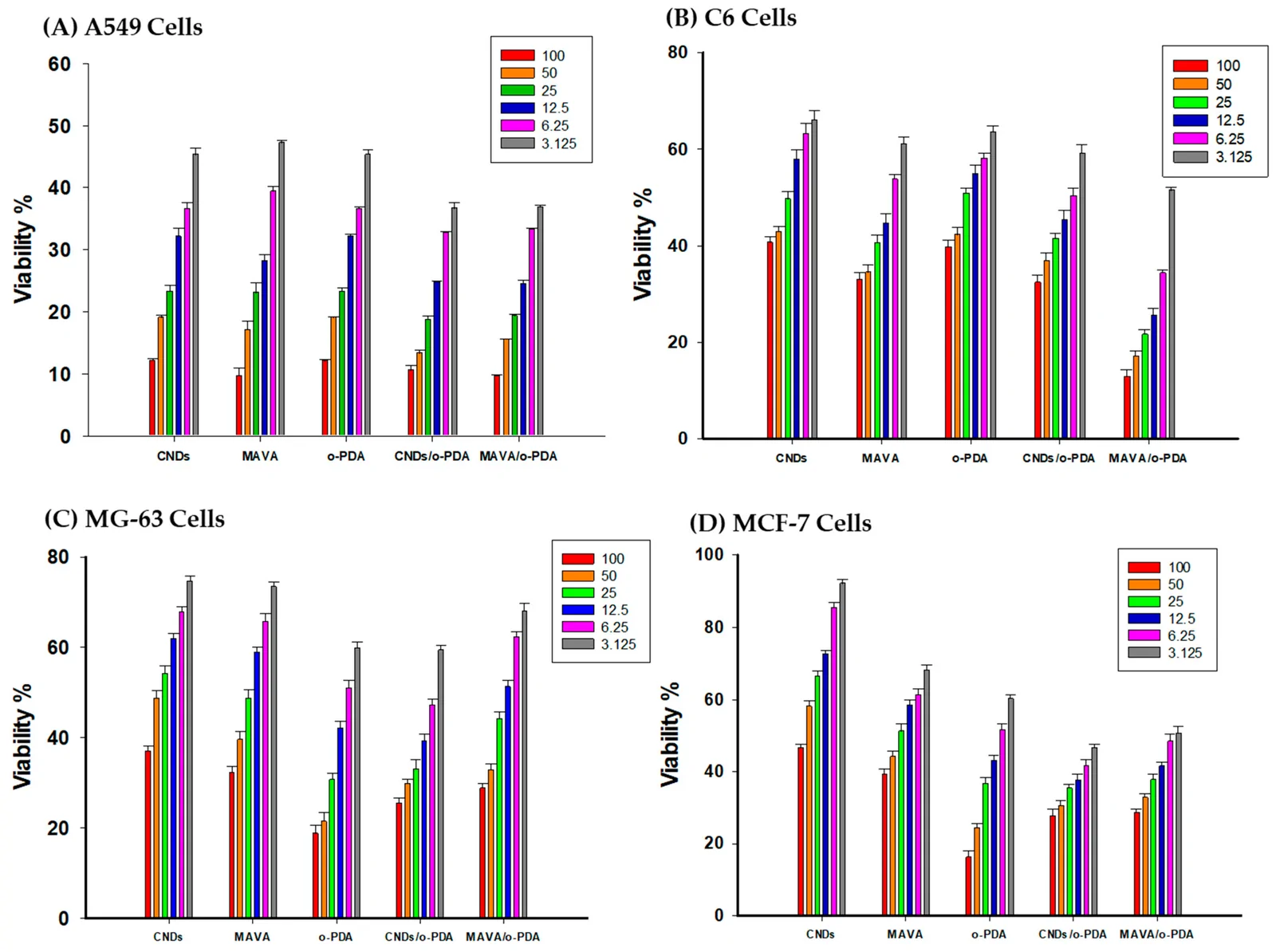
Cell viability of CNDs, *o*-PDA, CNDs/*o*-PDA, MAVA, and MAVA/*o*-PDA on A549 (**A**), C6 (**B**), MG-63 (**C**), and MCF-7 (**D**) cell lines.

At increasing concentrations, the MAVA/*o*-PDA and CNDs/*o*-PDA compounds significantly reduced cell viability in a dose-dependent manner in all cancer cell lines (*p* < 0.05) (Figure 2). These conjugates were more effective in MCF-7, MG-63, and A549 cancer cells compared to the use of MAVA, CNDs, and *o*-PDA alone. In C6 cells, a significant difference was observed in the 25–100 µg/mL concentration range, and the conjugates exhibited higher efficacy.

**Figure 3:**
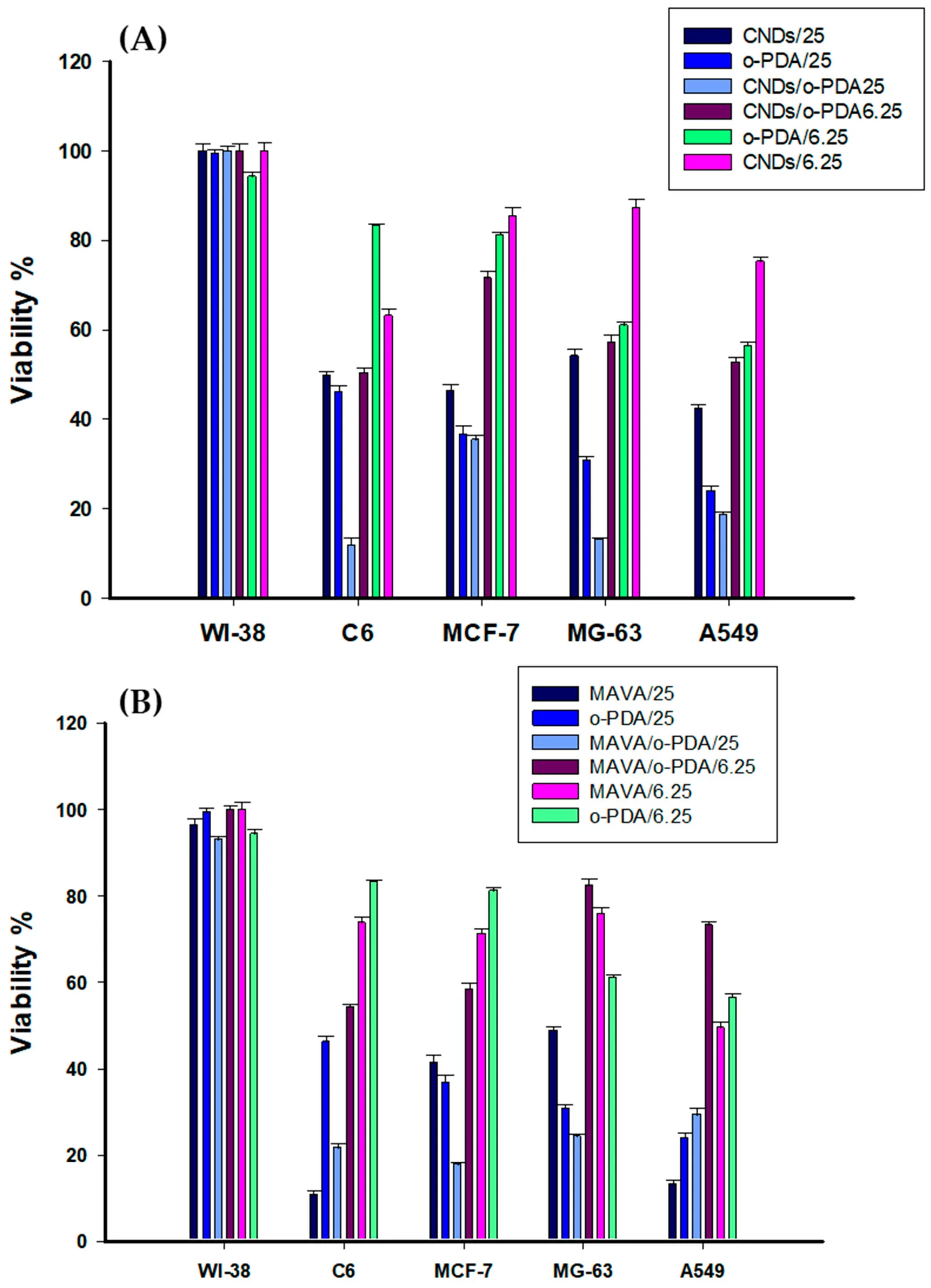
Comparative cell viability (%) of the conjugates compared to their individual components. (**A**) Effect of CNDs, *o*-PDA, and CNDs/*o*-PDA at concentrations of 25 and 6.25 µg/mL. (**B**) Effect of MAVA, *o*-PDA, and MAVA/*o*-PDA at the same concentrations. Data are presented as mean ± SD.

A similar trend is observed in both graphs. It was found that the conjugates caused a more pronounced decrease in viability in cancer cells compared to their individual components. While the effects on cell viability were more limited when CNDs and *o*-PDA were applied separately, the CNDs/*o*-PDA conjugate exhibited a stronger effect at the same concentrations. Similarly, the MAVA/*o*-PDA conjugate also caused a more pronounced loss of viability in cancer cells compared to MAVA or *o*-PDA alone (Figure 3). When both panels are evaluated together, it is evident that combining the components enhances cytotoxic activity. These results are consistent with the IC_50_ and selectivity index findings. Furthermore, it suggests that *o*-PDA contributes to increased anticancer activity when used in combination with both CNDs and MAVA.

**Figure 4:**
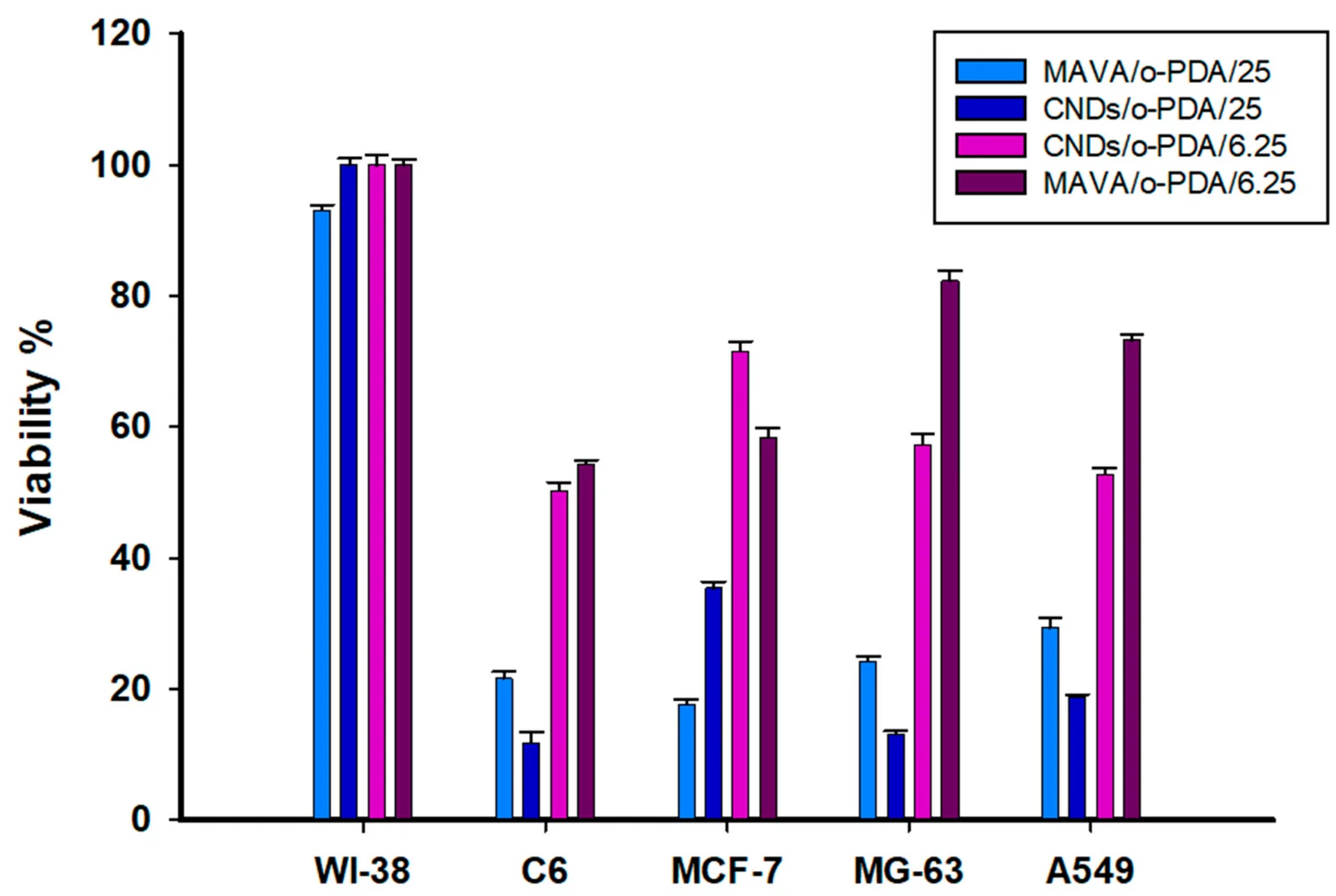
Cell viability (%) in WI-38, C6, MCF-7, MG-63, and A549 cells treated with CNDs/*o*-PDA and MAVA/*o*-PDA conjugates at concentrations of 25 and 6.25 µg/mL. Data are presented as mean ± SD.

While both conjugates significantly reduced cancer cell viability at 25 µg/mL, WI-38 cells were largely unaffected at this concentration. At 6.25 µg/mL, viability increased in all cell lines, although the increase varied by cell line. In particular, it was observed that viability in C6 cells remained lower than in other cell lines even at low concentrations (Figure 4). This result supports the strong efficacy of MAVA/*o*-PDA against C6 cells. Overall, it was observed that both conjugates reduced cell viability as the concentration increased and maintained their effect on cancer cells without causing significant toxicity in normal WI-38 cells.

### 3.3. Antimicrobial Activity Results

The MIC values for the compounds, determined in experiments conducted to evaluate their antimicrobial activity against bacteria and fungi, are presented in Table 3. The MIC values for the standard antibiotics used in the experiment (Amoxicillin/Clavulanic acid, Amikacin, Amoxicillin, Vancomycin, Fluconazole) and the MIC ranges reported in reference sources [Effective (MIC < 100 µg/mL), Moderate (100 < MIC ≤ 625 µg/mL), and Weak (MIC > 625 µg/mL)] were compared with the MIC values of the compounds [44,45].

When Gram-negative bacteria were evaluated as a whole, it was observed that the CNDs/*o*-PDA and MAVA/*o*-PDA compounds achieved significantly more effective MIC values compared to their individual components, CNDs, *o*-PDA, and MAVA. In particular, the antibacterial effect of CNDs/*o*-PDA on *E. coli* (MIC = 25 µg/mL) is even lower than the MIC value of the standard antibiotic (MIC = 32 µg/mL). When Gram-positive bacteria were evaluated as a whole, although it was demonstrated that the CNDs/*o*-PDA and MAVA/*o*-PDA compounds were more effective than their individual components, their MIC values remained above those of the reference antibiotics; thus, it was shown that the conjugates did not achieve the same level of efficacy as the reference antibiotics in this group of bacteria [29,30]. However, according to classification criteria widely used in the literature, MIC values are categorized as effective (MIC ≤ 100 µg/mL), moderate (100 < MIC ≤ 625 µg/mL), and weak (MIC > 625 µg/mL). Based on this classification, it was observed that both synthesized compounds (CNDs/*o*-PDA and MAVA/*o*-PDA) and their components exhibited effective MIC values against *E. faecalis and B. cereus* and moderately effective MIC values against *S. mutans*. Similarly, the antifungal activity of the synthesized compounds against *C. albicans* was well below the MIC values of the reference antibiotics; however, this is due to the fact that the antifungal agent used as a reference itself has an extremely low MIC profile. When evaluated according to general classification criteria in the literature, the synthesized compounds were found to exhibit moderate antifungal activity against *C. albicans* [44,45] (Table 3).

### 3.4. In Silico Analysis Results

#### 3.4.1. Molecular Docking Analysis Results

**Figure 5:**
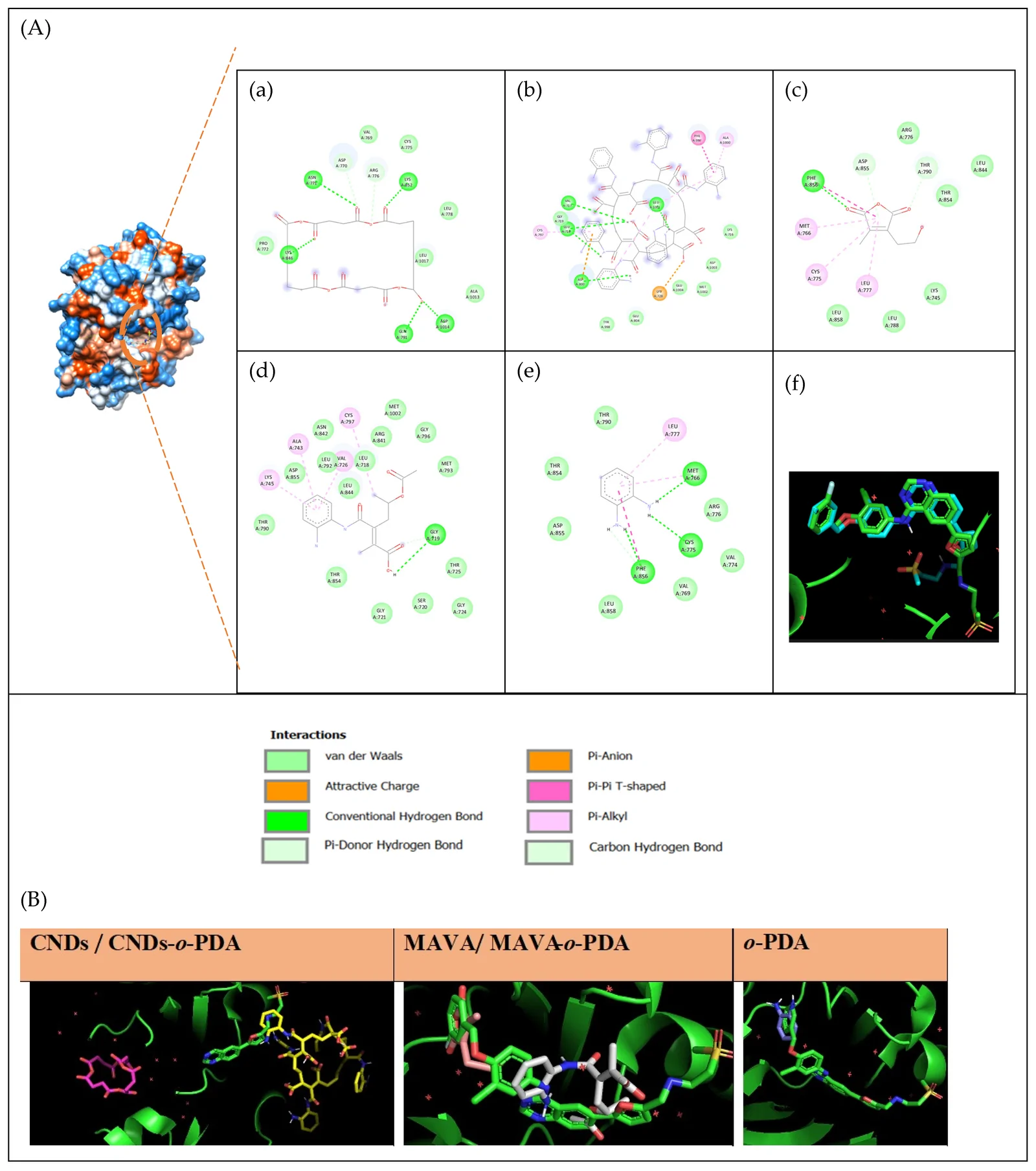
Molecular docking interactions and binding-site visualization of the investigated compounds against Epidermal Growth Factor Receptor (EGFR). (**A**) Molecular docking interactions of CNDs (**a**), CNDs/*o*-PDA (**b**), MAVA(**c**), MAVA/*o*-PDA (**d**), *o*-PDA (**e**), and the native ligand (**f**) against EGFR. (**B**) Visualization of the binding sites of CNDs/CNDs-*o*-PDA, MAVA/MAVA-*o*-PDA, and *o*-PDA for binding-region analysis. MAVA/MAVA-*o*-PDA, and *o*-PDA for binding-region analysis.

**Figure 6:**
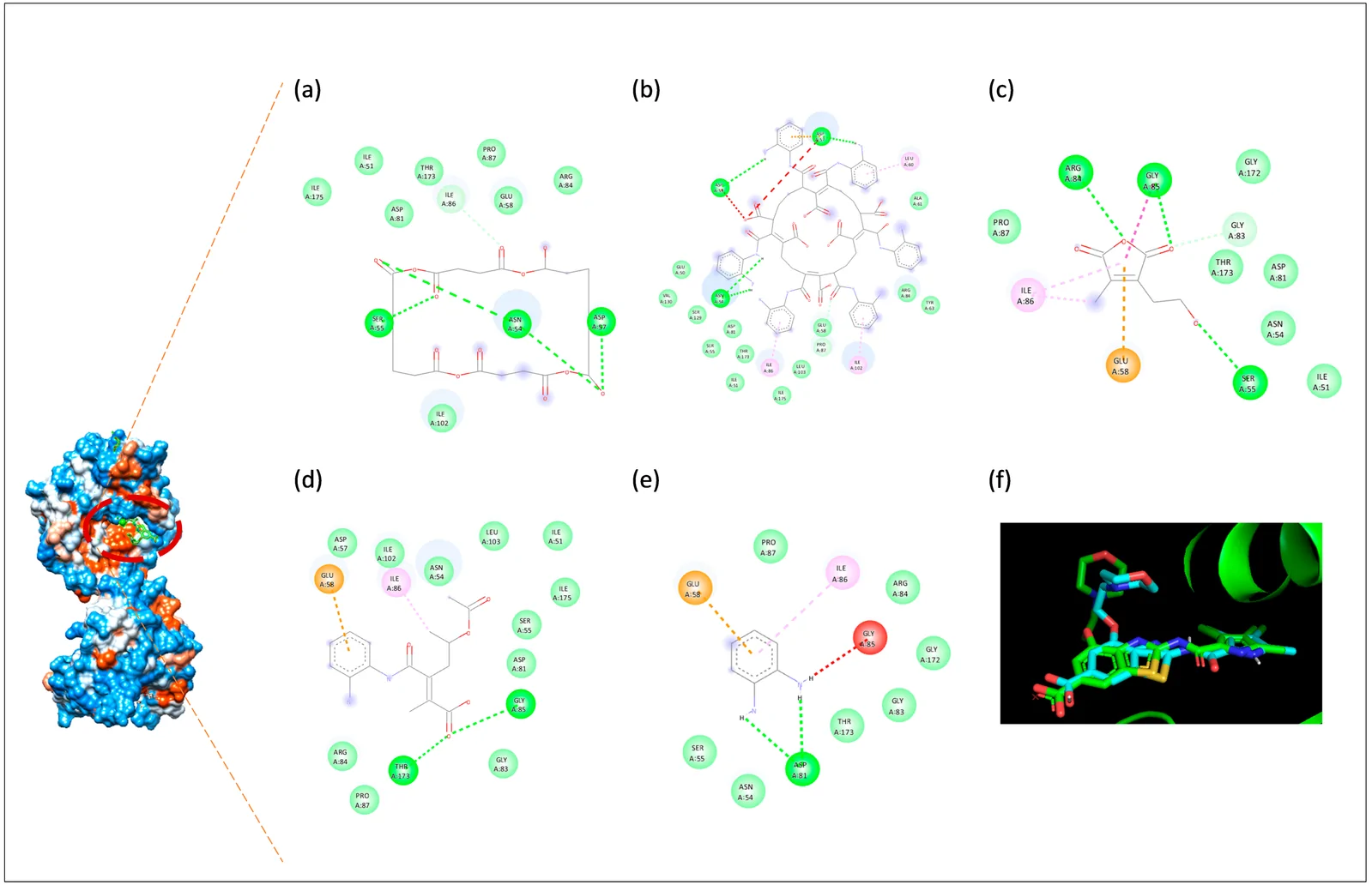
Molecular docking interactions of CNDs (**a**), CNDs/*o*-PDA (**b**), MAVA (**c**), MAVA/*o*-PDA (**d**), *o*-PDA (**e**), and native ligand (**f**) against *Staphylococcus aureus*.

**Figure 7:**
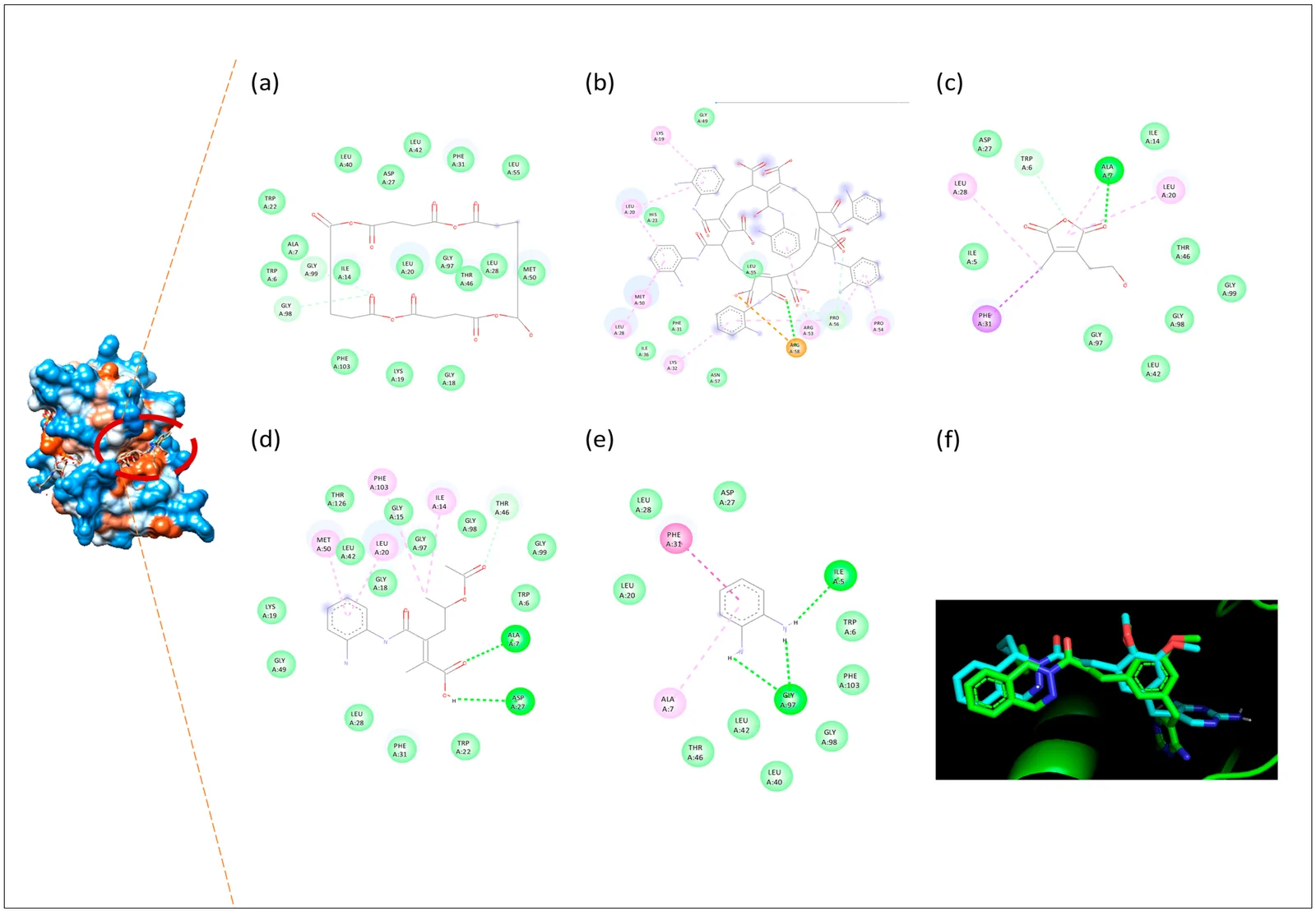
Molecular docking interactions of CNDs (**a**), CNDs/*o*-PDA (**b**), MAVA (**c**), MAVA/*o*-PDA (**d**), *o*-PDA (**e**), and native ligand (**f**) against *Enterococcus faecalis*.

**Figure 8:**
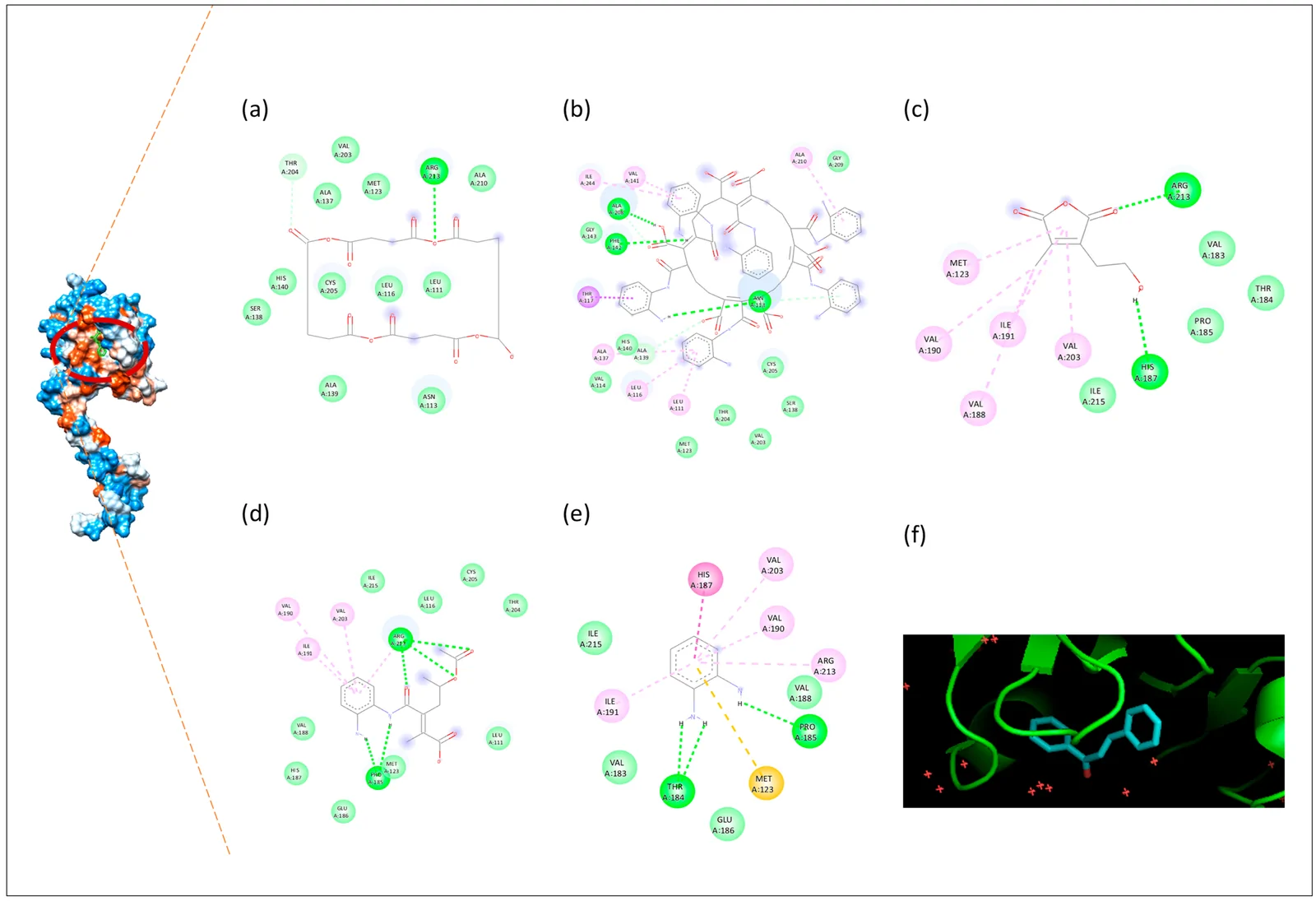
Molecular docking interactions of CNDs (**a**), CNDs/*o*-PDA (**b**), MAVA (**c**), MAVA/*o*-PDA (**d**), *o*-PDA (**e**), and native ligand (**f**) against *Streptococcus mutans*.

**Figure 9:**
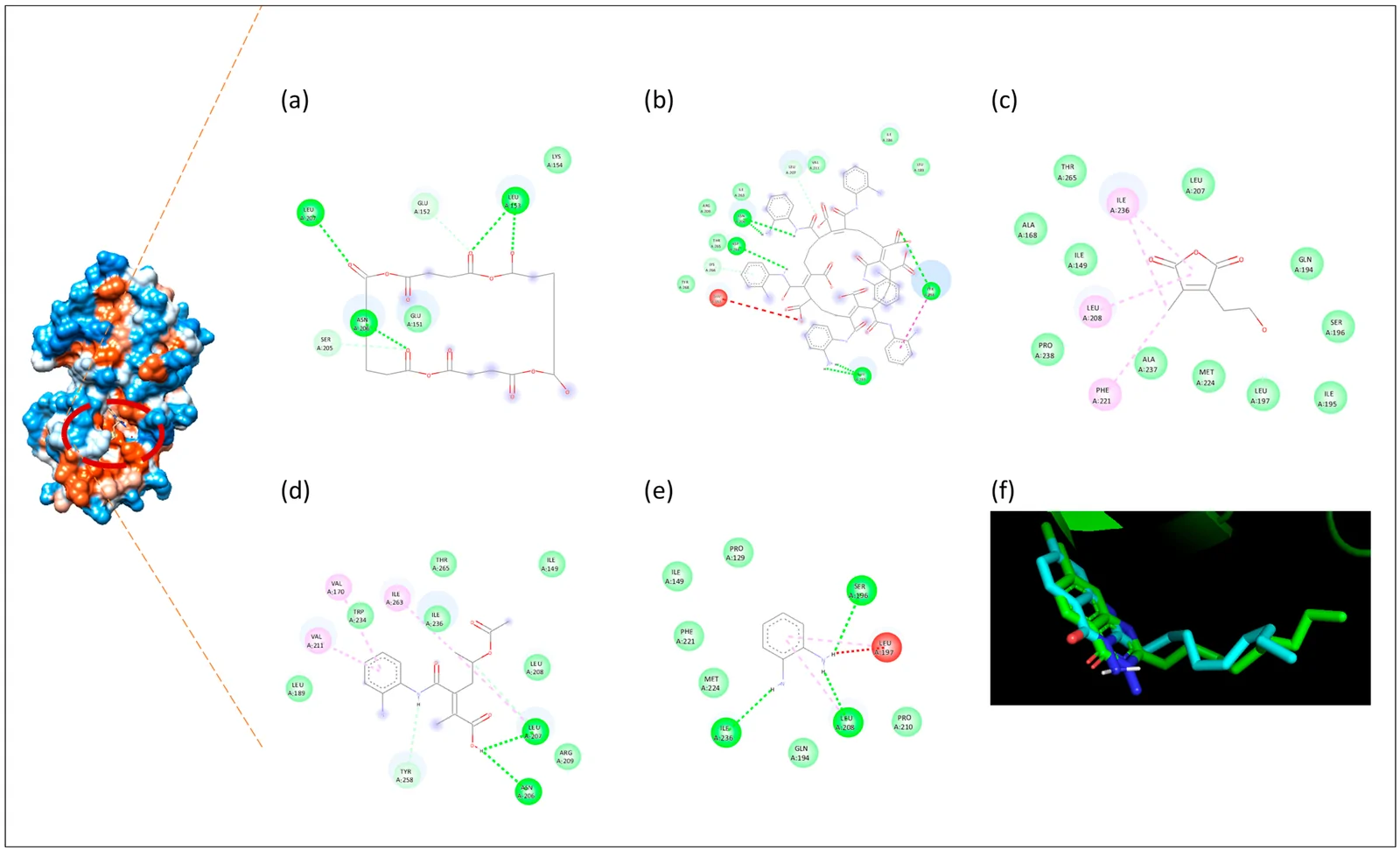
Molecular docking interactions of CNDs (**a**), CNDs/*o*-PDA (**b**), MAVA (**c**), MAVA/*o*-PDA (**d**), *o*-PDA (**e**), and native ligand (**f**) against *Pseudomonas aeruginosa*.

**Figure 10:**
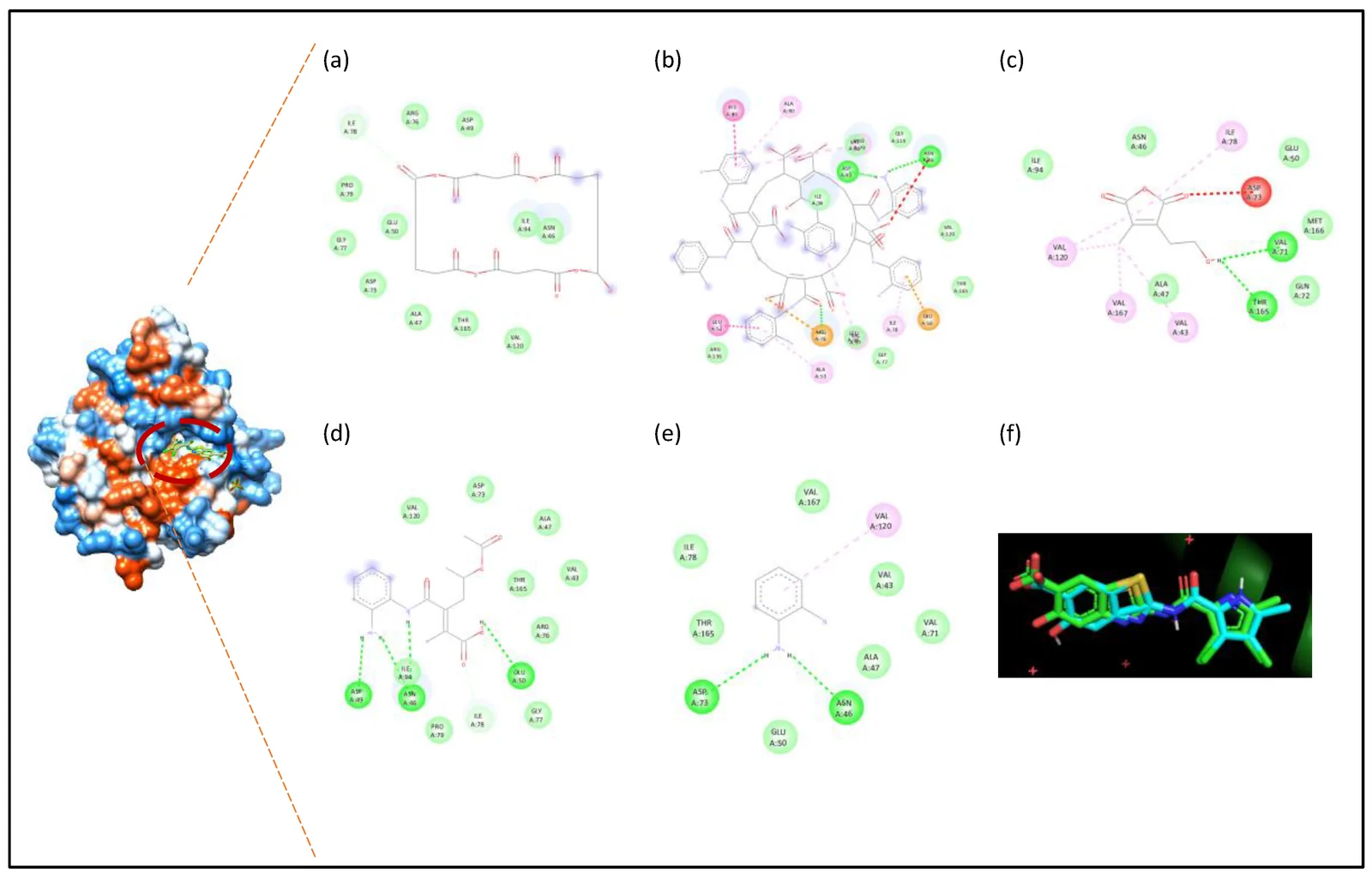
Molecular docking interactions of CNDs (**a**), CNDs/*o*-PDA (**b**), MAVA (**c**), MAVA/*o*-PDA (**d**), *o*-PDA (**e**), and native ligand (**f**) against *Escherichia coli*.

**Figure 11:**
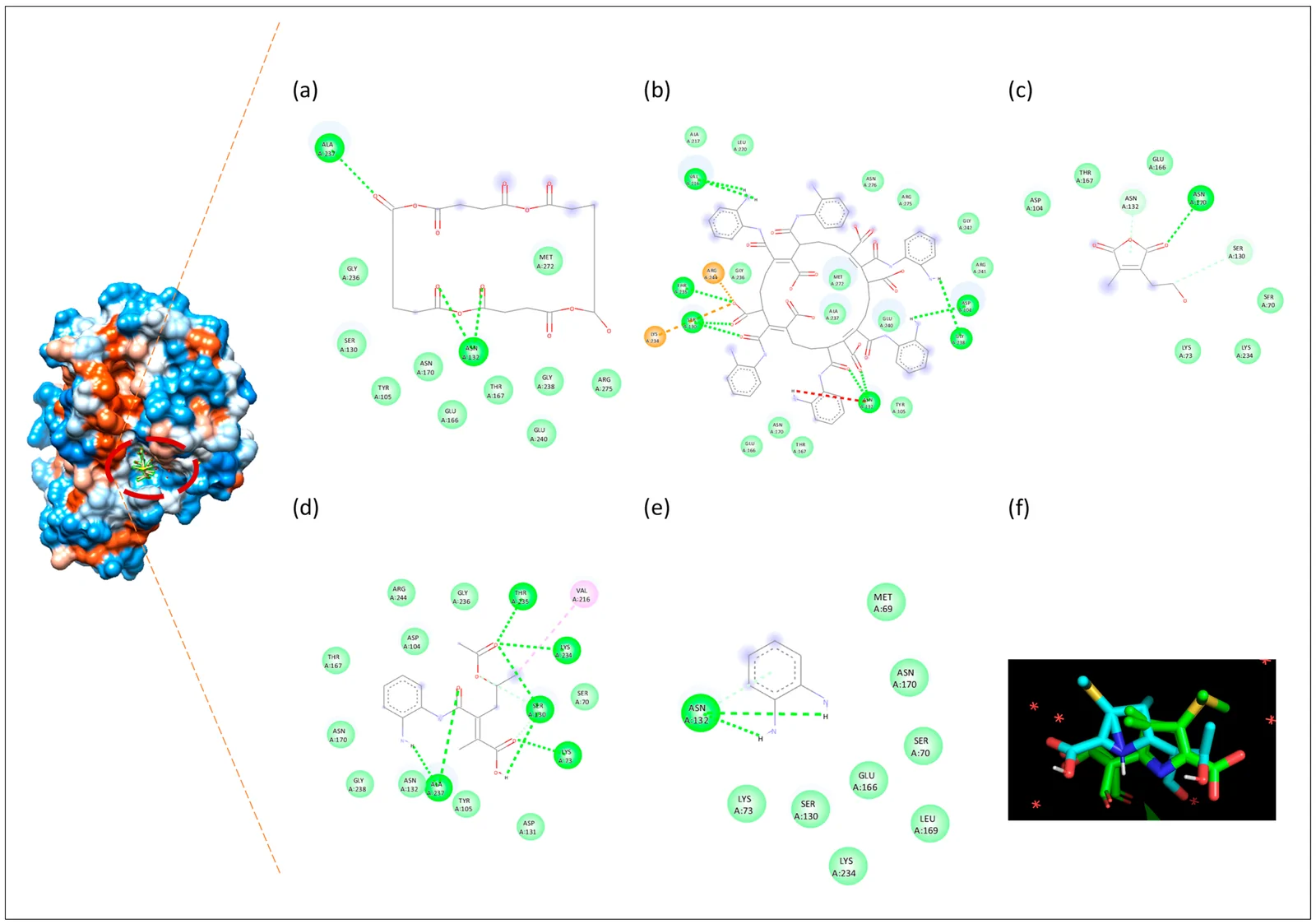
Molecular docking interactions of CNDs (**a**), CNDs/*o*-PDA (**b**), MAVA (**c**), MAVA/o-PDA (**d**), *o*-PDA (**e**), and native ligand (**f**) against *Klebsiella pneumoniae*.

**Figure 12:**
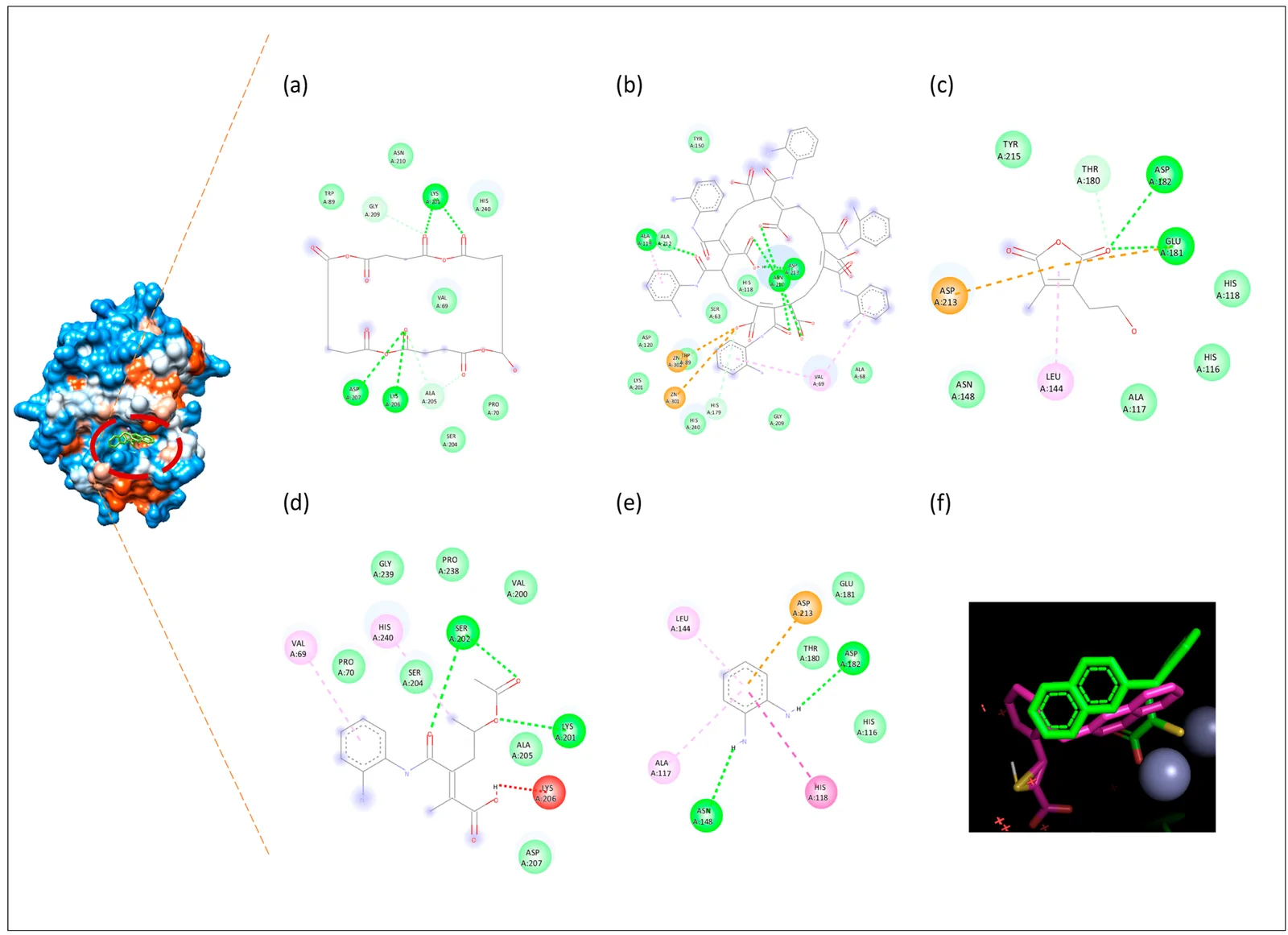
Molecular docking interactions of CNDs (**a**), CNDs/*o*-PDA (**b**), MAVA (**c**), MAVA/*o*-PDA (**d**), *o*-PDA (**e**), and native ligand (**f**) against *Bacillus cereus*.

**Figure 13:**
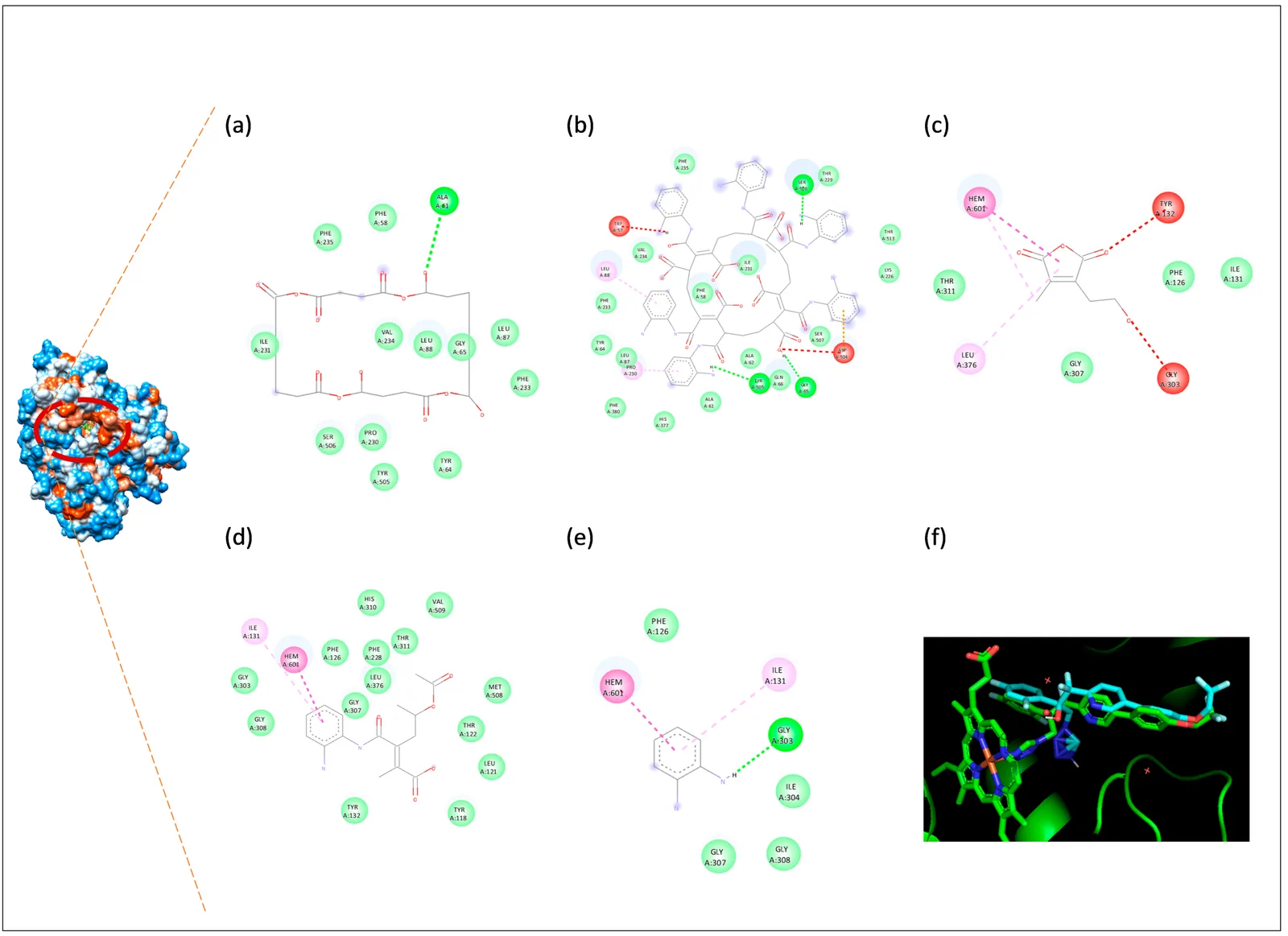
Molecular docking interactions of CNDs (**a**), CNDs/*o*-PDA (**b**), MAVA (**c**), MAVA/*o*-PDA (**d**), *o*-PDA (**e**), and native ligand (**f**) against *Candida albicans*.

Molecular docking analyses were performed to investigate the potential anticancer and antimicrobial activities of the evaluated compounds and to characterize their binding modes with the selected target proteins. The reliability of the molecular docking protocol was validated by redocking the co-crystallized reference ligand into the corresponding target binding site. The obtained results were visualized by superimposing the ligands, and the binding poses of the original crystal ligand and the redocked ligand were compared for each target. In addition, the binding energies and interaction modes of two *o*-PDA derivatives and other free compounds (CNDs, *o*-PDA, CNDs/*o*-PDA, MAVA, and MAVA/*o*-PDA) with the target proteins were investigated to evaluate their binding affinities and molecular interaction profiles. Overall, the redocking results indicated that the docking protocol was reliable, as the redocked ligands reproduced binding poses similar to those of the co-crystallized ligands (Figure 5f, Figure 6f, Figure 7f, Figure 8f, Figure 9f, Figure 10f, Figure 11f, Figure 12f and Figure 13f). In some molecules, particularly those containing Zn and Fe, failure to obtain binding poses similar to the crystal structure during redocking may be because docking score functions do not model metal-centered interactions accurately enough. Furthermore, the coordination geometry of metal ions and minor changes in the active site environment may also lead to this mismatch.

#### 3.4.2. Molecular Docking Analysis of Anticancer Activity

The anticancer activity of the investigated compounds was evaluated by molecular docking against the selected target proteins. Docking scores for EGFR: CNDs (−7.9 kcal/mol), *o*-PDA (−5.1 kcal/mol), CNDs/*o*-PDA (−5.3 kcal/mol), MAVA (−5.6 kcal/mol), MAVA/*o*-PDA (−6.6 kcal/mol), and −11.6 kcal/mol for the co-crystal ligand (Table 4). In general, whilst *o*-PDA on its own exhibited a lower docking score, higher docking scores were observed when it formed complexes with CNDs and MAVA.

The 2D interaction diagrams, redocking results, and binding site images of the docked molecules are presented in Figure 5A.

To further investigate the binding modes and binding site localization of the compounds, their three-dimensional binding poses were visualized. In some cases, while CNDs alone did not bind effectively within the active site, the CNDs/*o*-PDA derivative was observed to bind in a position similar to that of the reference ligand (Figure 5B). A similar trend was also observed for the MAVA and MAVA/*o*-PDA derivatives (Supplementary File, Figures S2–S9). In addition, differences in the interaction profiles of the investigated compounds were observed across the target proteins. In some cases, the complex forms exhibited more favorable interaction patterns than the corresponding individual compounds, whereas such an effect was not consistently observed for all targets. These findings indicate that the binding behavior of the investigated compounds may vary depending on the structural characteristics of the target binding site.

#### 3.4.3. Molecular Docking Analysis of Antimicrobial Activity

The antimicrobial molecular docking results of CNDs, *o*-PDA, CNDs/*o*-PDA, MAVA, and MAVA/*o*-PDA against the selected microbial targets are presented in Table 5. For *Escherichia coli*, the binding energies ranged from −6.9 to −5.0 kcal/mol; for *Klebsiella pneumoniae*, from −7.3 to −4.5 kcal/mol; and for *Pseudomonas aeruginosa*, from −6.7 to −4.9 kcal/mol. The binding energies for *Staphylococcus aureus* ranged from −7.2 to −4.9 kcal/mol, while those for *Enterococcus faecalis* ranged from −7.7 to −5.0 kcal/mol. For *Streptococcus mutans* and *Bacillus cereus*, the binding energies ranged from −7.3 to −4.4 kcal/mol and −7.3 to −4.6 kcal/mol, respectively. For *Candida albicans*, the binding energies ranged from −8.0 to −5.2 kcal/mol.

The 2D interaction profiles of the ligands with the selected microbial targets are presented in Figure 6, Figure 7, Figure 8, Figure 9, Figure 10, Figure 11, Figure 12 and Figure 13, while the corresponding 3D interaction views (Figures S2–S9) and the color legend for the different types of molecular interactions (Table S1) are provided in the Supplementary File.

For *S. aureus*, CNDs have been shown to form hydrogen bonds and carbon–hydrogen bond interactions with the amino acids ASN54, SER55, ASP57, and ILE86. Conventional hydrogen bonds were identified with ASN54, SER55, and ASP57, while carbon–hydrogen bond interactions were identified with ASN54 and ILE86. The CNDs/*o*-PDA complex exhibited conventional hydrogen bonds with ASN54, ASP53, and ASP57, and a carbon–hydrogen bond interaction with PRO87. Additionally, π-anion interactions with ASP57 and π-alkyl hydrophobic interactions with ILE86, LEU60, and ILE102 were identified. The MAVA ligand exhibited conventional hydrogen bonding with SER55, ARG84, and GLY85, as well as a carbon–hydrogen bond interaction with GLY83. Furthermore, π-anion interactions with GLU58, amide-π interactions with GLY85/ILE86, and alkyl and π-alkyl hydrophobic interactions with ILE86 were identified. The MAVA/*o*-PDA complex exhibited conventional hydrogen bonding with GLY85 and THR173. Furthermore, an electrostatic π-anion interaction with GLU58 and an alkyl hydrophobic interaction with ILE86 were identified. The *o*-PDA ligand exhibited a conventional hydrogen bond interaction with ASP81. Additionally, an electrostatic π-anion interaction with GLU58 and a π-alkyl hydrophobic interaction with ILE86 were identified.

For *E. faecalis*, CNDs exhibited carbon–hydrogen bond interactions with the amino acids GLY98 and GLY99. The CNDs/*o*-PDA complex exhibited a conventional hydrogen bond and electrostatic attraction with ARG58, as well as a carbon–hydrogen bond interaction with PRO56. In addition, π-alkyl hydrophobic interactions were identified with PRO54, PRO56, LYS32, LEU20, LEU28, MET50, LYS19, and ARG53. The MAVA ligand exhibited a conventional hydrogen bond with ALA7 and a carbon–hydrogen bond interaction with TRP6. Furthermore, π-sigma interactions with PHE31, alkyl interactions with LEU28, and π-alkyl hydrophobic interactions with ALA7 and LEU20 were identified. The MAVA/*o*-PDA complex exhibited conventional hydrogen bonding with ALA7 and ASP27, as well as a carbon–hydrogen bond interaction with THR46. Furthermore, alkyl hydrophobic interactions with ILE14 and π-alkyl hydrophobic interactions with PHE103, LEU20, and MET50 were identified. The *o*-PDA ligand exhibited conventional hydrogen bond interactions with ILE5 and GLY97. Additionally, a π-π-stacked interaction with PHE31 and a π-alkyl hydrophobic interaction with ALA7 were identified.

For *S. mutans,* the CNDs ligand exhibited a conventional hydrogen bond with ARG213 and a carbon–hydrogen bond interaction with THR204. The CNDs/*o*-PDA complex for *S. mutans* exhibited conventional hydrogen bonds with ASN113, PHE142, and ALA208; and carbon–hydrogen bond interactions with ALA139 and ALA208. Additionally, π-donor hydrogen bonding with ASN113, π-sigma interactions with THR117, and π-alkyl hydrophobic interactions with LEU111, LEU116, ALA137, VAL141, ILE244, and ALA210 were identified. The MAVA ligand exhibited conventional hydrogen bond interactions with ARG213 and HIS187. Furthermore, alkyl interactions were identified with VAL188, VAL190, and ILE191, and π-alkyl hydrophobic interactions were identified with MET123, ILE191, and VAL203. The MAVA/*o*-PDA complex exhibited conventional hydrogen bonding with ARG213 and PRO185, as well as a carbon–hydrogen bond interaction with ARG213. Furthermore, π-alkyl hydrophobic interactions were identified with VAL190, ILE191, VAL203, and ARG213. The *o*-PDA ligand exhibited conventional hydrogen bonding with PRO185 and THR184. Furthermore, π-sulfur interactions with MET123, amide-π stacking with HIS187/VAL188, and π-alkyl hydrophobic interactions with VAL190, ILE191, VAL203, and ARG213 were identified.

For *P. aeruginosa*, the CNDs ligand exhibited conventional hydrogen bonding with LEU153, ASN206, and LEU207, and carbon–hydrogen bonding interactions with GLU152 and SER205. The CNDs/*o*-PDA complex exhibited conventional hydrogen bonding with TYR258, GLU259, ASN206, and ASP264, as well as a carbon–hydrogen bond interaction with LEU207. Additionally, a π-donor hydrogen bond and a π-alkyl hydrophobic interaction with LYS266, as well as a π-π stacked interaction with TYR258, were identified. The MAVA ligand exhibited alkyl interactions with ILE236 and π-alkyl hydrophobic interactions with PHE221, LEU208, and ILE236. The MAVA/*o*-PDA complex exhibited conventional hydrogen bonding with ASN206 and LEU207, as well as a carbon–hydrogen bond interaction with LEU207. Additionally, a π-donor hydrogen bond with TYR258, alkyl interactions with LEU207 and ILE263, and π-alkyl hydrophobic interactions with VAL170 and VAL211 were identified. The *o*-PDA ligand exhibited conventional hydrogen bond interactions with SER196, LEU208, and ILE236. Furthermore, π-alkyl hydrophobic interactions with LEU197 and LEU208 were identified.

The CNDs ligand for *E. coli* exhibited a carbon–hydrogen bond interaction with ILE78. The CNDs/*o*-PDA complex for *E. coli* exhibited a conventional hydrogen bond and electrostatic attraction with ARG76, as well as conventional hydrogen bond interactions with ASP49 and ASN46. In addition, electrostatic π-anion interactions with GLU50, π-π T-shaped interactions with HIS83, amide-π stacked interactions with LEU52/ALA53, and π-alkyl hydrophobic interactions with ILE78, ALA53, PRO79, ALA90, and VAL97 were identified. The MAVA ligand exhibited conventional hydrogen bonding interactions with VAL71 and THR165. Furthermore, alkyl interactions were identified with VAL43, VAL120, and VAL167, and π-alkyl hydrophobic interactions were identified with ILE78 and VAL120. The MAVA/*o*-PDA complex exhibited conventional hydrogen bonding with ASN46, ASP49, and GLU50, as well as a carbon–hydrogen bond interaction with ILE78. The *o*-PDA ligand exhibited conventional hydrogen bonding with ASN46 and ASP73. In addition, a π-alkyl hydrophobic interaction with VAL120 was identified.

The CNDs ligand for *K. pneumoniae* exhibited conventional hydrogen bonding interactions with ASN132 and ALA237. The CNDs/*o*-PDA complex exhibited conventional hydrogen bonding and electrostatic interactions with ARG244, and electrostatic interactions with LYS234. In addition, conventional hydrogen bond interactions with SER130, ASN132, and THR235; conventional hydrogen bond interactions with ASP104, GLY238, and VAL216; and a carbon–hydrogen bond interaction with SER130 were identified. The MAVA ligand exhibited conventional hydrogen bonding with ASN170; carbon–hydrogen bonding with SER130; and π-donor hydrogen bonding with ASN132. The MAVA/*o*-PDA complex exhibited conventional hydrogen bond interactions with LYS73, SER130, LYS234, THR235, and ALA237, as well as a carbon–hydrogen bond interaction with SER130. In addition, an alkyl-hydrophobic interaction with VAL216 was identified. The *o*-PDA ligand exhibited conventional hydrogen bond and π-donor hydrogen bond interactions with ASN132.

For *B. cereus*, CNDs exhibited conventional hydrogen bonding with the ligands LYS201, LYS206, and ASP207, and carbon–hydrogen bonding interactions with ALA205 and GLY209. The CNDs/*o*-PDA complex exhibited electrostatic attraction with Zn301 and Zn302; conventional hydrogen bonding with ALA119 and ASN210; conventional hydrogen bonding with ASP213; and carbon–hydrogen bonding with HIS179. In addition, π-alkyl hydrophobic interactions were observed with VAL69 and ALA119. The MAVA ligand exhibited conventional hydrogen bonding with GLU181 and ASP182, as well as a carbon–hydrogen bond interaction with THR180. Furthermore, electrostatic π-anion interactions with GLU181 and ASP213, and π-alkyl hydrophobic interactions with LEU144 were identified. The MAVA/*o*-PDA complex exhibited conventional hydrogen bond interactions with LYS201 and SER202. Additionally, π-alkyl hydrophobic interactions with HIS240 and VAL69 were identified. The *o*-PDA ligand exhibited conventional hydrogen-bonding interactions with ASP182 and ASN148. Furthermore, electrostatic π-anion interactions with ASP213, π-π-stacked interactions with HIS118, and π-alkyl hydrophobic interactions with ALA117 and LEU144 were identified.

For *C. albicans*, the CNDs ligand exhibited conventional hydrogen bonding with ALA61. The CNDs/*o*-PDA complex exhibited conventional hydrogen bonding with SER506, TYR505, and GLY65. In addition, electrostatic π-anion interactions with ASP504 and π-alkyl hydrophobic interactions with PRO230 were identified. The MAVA ligand exhibited π-π-stacked and π-alkyl hydrophobic interactions with HEM601, and π-alkyl hydrophobic interactions with LEU376. The MAVA/*o*-PDA complex exhibited π-π-stacked and π-alkyl hydrophobic interactions with HEM601 and ILE131, respectively. The *o*-PDA ligand exhibited a conventional hydrogen bond interaction with GLY303. Furthermore, π-π-stacked and π-alkyl hydrophobic interactions with HEM601 and ILE131, respectively, were identified.

#### 3.4.4. ADMET and Drug-Likeness Analysis

**Figure 14:**
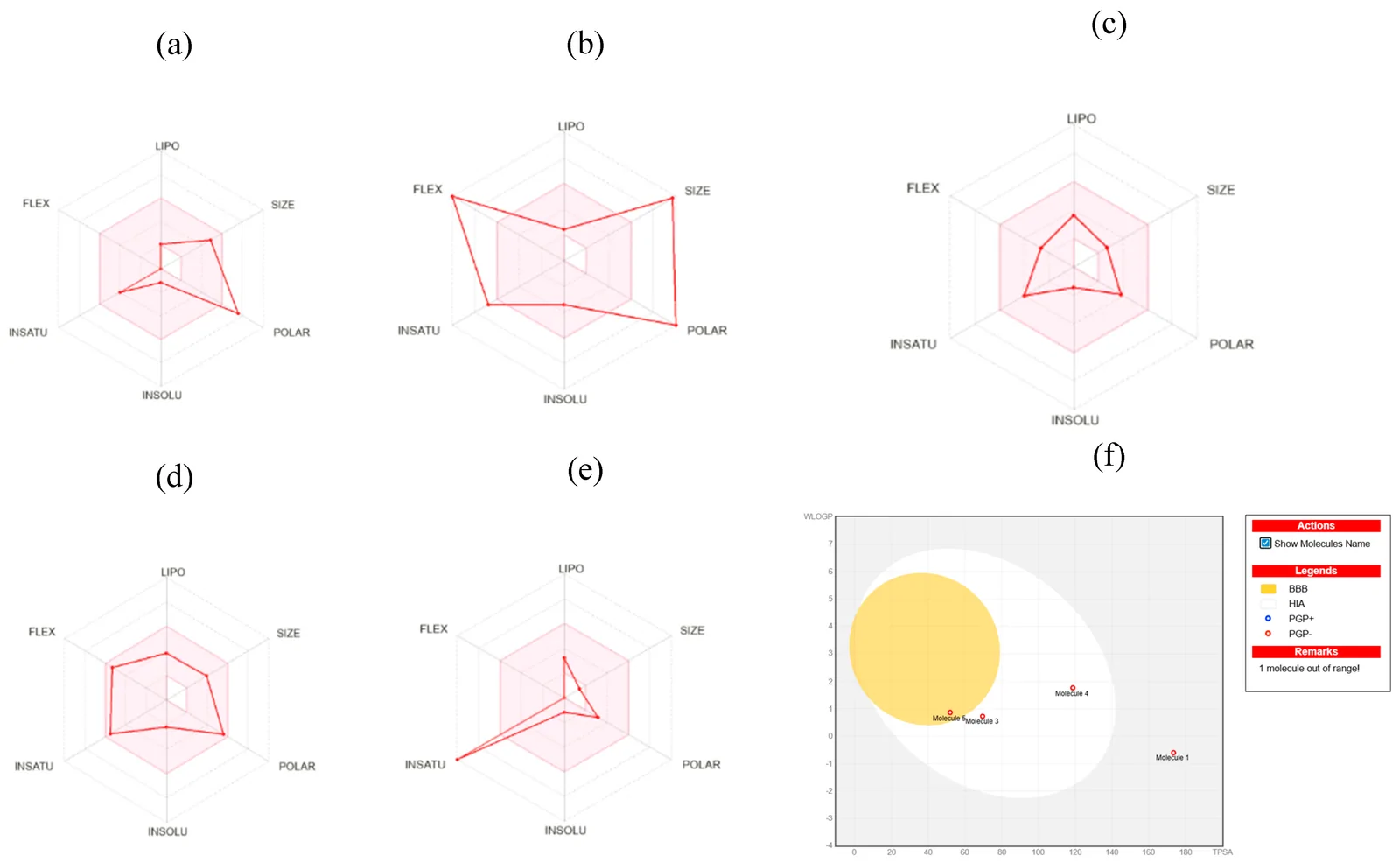
Bioavailability radar plots of CNDs (**a**), CNDs/o-PDA (**b**), MAVA (**c**), MAVA/o-PDA (**d**), and *o*-PDA (**e**), and the Boiled-Egg model for the test compounds (**f**).

As part of the SwissADME analysis, radar plots and the boiled egg model were generated for five compounds (Figure 14). Based on the results obtained, it was determined that two compounds (MAVA and MAVA-*o*-PDA) exhibited high gastrointestinal (GI) absorption, whilst one compound (CNDS-*o*-PDA) fell outside the recommended ranges for both GI absorption and blood–brain barrier (BBB) permeability. Furthermore, it was observed that the *o*-PDA compound was the only molecule capable of crossing the blood–brain barrier.

In general, the most pronounced distinction between molecules is observed between the CNDs/CNDs-*o*-PDA derivatives and the MAVA/MAVA-*o*-PDA/*o*-PDA group. When the CNDs and CNDs-*o*-PDA derivatives are evaluated individually, the number of rotatable bonds is 0 in CNDs, whereas this value rises to 16 in CNDs-*o*-PDA. Whilst the number of hydrogen bond acceptors remains at 12 in both compounds, the number of hydrogen bond donors has increased from 0 in CNDs to 12 in CNDs-*o*-PDA. Upon examination of the TPSA values, it is observed that the polar surface area, which is 173.48 Å^2^ in CNDs, has increased to 369.68 Å^2^ in CNDs-*o*-PDA. When evaluating the MAVA derivatives, the TPSA is 69.67 Å^2^ in MAVA, whilst this value rises to 118.72 Å^2^ in MAVA-o-PDA. Similarly, whilst the number of rotatable bonds in MAVA is 4, the number of hydrogen bond acceptors is 5, and the number of hydrogen bond donors is 0, in the MAVA-*o*-PDA derivative these values increase to 8, 5, and 3, respectively. *o*-PDA, with a TPSA value of 52.04 Å^2^, 0 rotatable bonds, and 2 hydrogen bond donors, stands out as the smallest structure with the lowest polarity. In terms of gastrointestinal absorption, CNDs and CNDs-*o*-PDA exhibit low gastrointestinal absorption, whilst MAVA, MAVA-*o*-PDA and *o*-PDA demonstrate high gastrointestinal absorption. Furthermore, it has been determined that the *o*-PDA compound is the only molecule capable of crossing the blood–brain barrier (BBB).

Whilst the MAVA and MAVA-*o*-PDA compounds exhibit lipophilic properties with positive LogP values (1.14 and 1.38), *o*-PDA displays moderate lipophilicity with a value of 0.61. In contrast, the CNDs and CNDs/*o*-PDA derivatives exhibit hydrophilic character with negative LogP values (−0.35 and −0.78) (Table 6).

When drug similarity rules are assessed, *o*-PDA, MAVA, and MAVA-*o*-PDA show no Lipinski violations, whereas CNDs deviate significantly from the rules with just one violation (HBA > 10) and CNDs-*o*-PDA with three violations (MW > 500, HBA > 10, HBD > 5). This is also reflected in the bioavailability scores; whilst CNDs-*o*-PDA has a very low value (0.11), the other molecules demonstrate moderate bioavailability potential in the range of 0.55–0.56 (Table 7).

An analysis of the ProTox 3.0 prediction results revealed that the CNDs and CNDs-*o*-PDA compounds were classified as toxicity class 6, whereas MAVA and *o*-PDA were predicted to be toxicity class 4, and MAVA-*o*-PDA was predicted to be toxicity class 5. These results indicate that CND derivatives possess a lower potential for acute toxicity, whilst MAVA and *o*-PDA derivatives may carry a relatively higher risk of toxicity.

## 4. Discussion

Surface functionalization processes have yielded highly promising results, particularly in recent years, for the development of candidate molecules in the biomedical field. Here, for surface functionalization, the same anhydride functionality—characterized by two acyl groups bonded to a single oxygen atom and having the general formula R-C(=O)-O-C(=O)-R’—is present in such a way that it exhibits a uniform distribution on the surfaces of both MAVA, selected as the polymer, and CNDs, selected as quantum dots. As such, this anhydride ring is particularly suitable for conjugation with small drugs and other pharmaceutically active molecules possessing a primary amino (-NH_2_) functional group. Conjugation occurs readily under mild conditions via a ring-opening reaction, and the desired candidate molecules—referred to as conjugates—can be transformed into polymer/drug or quantum dot/drug pairs that are free from undesirable side effects and bioavailability-related drawbacks. The reason for selecting these two structures stems primarily from their chemical and surface properties: While the first structure selected as the carrier, MAVA, has a linear polymer chain, the second structure, CNDs, has a spherical structure. Consequently, the anhydride ring in question is distributed linearly in the former and regularly around a spherical surface in the latter. The main point here, from the perspective of steric hindrance, is determining which of these surfaces the structure selected as the drug’s active ingredient will bind to more effectively and efficiently, and how the resulting conjugates will interact with their targets (cancer and bacterial cells) once bound.

To evaluate the potential effects of these structural differences on the biological properties of the conjugates and their interactions with target molecules, this study utilized CNDs, *o*-PDA, and MAVA components, and the anticancer, antimicrobial, and physicochemical/pharmacokinetic properties of previously synthesized and characterized CNDs/*o*-PDA and MAVA/*o*-PDA conjugates were evaluated comparatively. The findings indicate that the conjugation process consistently enhances both anticancer and antimicrobial activity compared to the individual components.

The results of the anticancer activity assays demonstrated that the CNDs/*o*-PDA and MAVA/*o*-PDA conjugates achieved significantly lower IC_50_ values compared to their individual components across all cancer cell lines. This finding is consistent with other MAVA-based conjugate systems designed with methotrexate and gemcitabine; it has been reported that MAVA-MTX and MAVA-GEM conjugates exhibit higher anticancer activity and lower toxicity to normal cells compared to the free drugs in MCF-7 cells [12]. This latest finding aligns with the lowest IC_50_ value (6.50 µg/mL) achieved by MAVA/*o*-PDA in C6 cells in the present study, suggesting that MAVA-based conjugates exhibit consistent sensitivity against this cell line. A similar trend has been reported in studies using carbon quantum dot (CND)-based delivery systems; it has been shown that carbon quantum dots conjugated with doxorubicin provide stronger cytotoxicity and higher cellular uptake in MCF-7 cells compared to the free drug [46]; this suggests a mechanism similar to the increased efficacy of CNDs/*o*-PDA observed in the present study.

Since the cytotoxic effect of doxorubicin varies depending on the duration of treatment, the viability assay used, and the number of passages of the cell line, the IC_50_ values reported in the literature vary widely. In terms of selectivity, the SI values of doxorubicin were calculated as 5.24, 3.69, 4.06, and 7.51 for C6, MCF-7, MG-63, and A549 cells, respectively. The tested conjugates, particularly CNDs/*o*-PDA, exhibited higher SI values than doxorubicin in all cancer cell lines. MAVA and MAVA/*o*-PDA, on the other hand, were found to be more selective than doxorubicin in C6, MCF-7, and MG-63 cells, but less selective than doxorubicin in A549 cells. These findings suggest that conjugates may generally offer a more favorable selectivity profile between cancer cells and normal cells compared to standard chemotherapeutic agents.

However, it should be noted that the absolute cytotoxic potency of the test materials is lower than that of doxorubicin. [For example, the IC_50_ value of MAVA/*o*-PDA in C6 cells is 6.50 µg/mL, whereas that of doxorubicin is 1.82 µg/mL]. Therefore, the advantage of these compounds lies in their selectivity profile rather than their potency. Furthermore, since C6 cells are of rat origin and WI-38 cells are of human origin, the SI values calculated for C6 cells should be interpreted with caution due to interspecies differences. It should also be noted that our results pertain to in vitro conditions and that the safety and efficacy of these compounds must be validated through in vivo studies.

In molecular binding analyses conducted to support experimental anticancer findings, it was determined that the binding scores of the compounds against EGFR varied (CNDs: −7.9 kcal/mol; *o*-PDA: −5.1 kcal/mol; CNDs/*o*-PDA: −5.3 kcal/mol; MAVA: −5.6 kcal/mol; MAVA/*o*-PDA: −6.6 kcal/mol; co-crystal ligand: −11.6 kcal/mol). Although CNDs alone yielded the lowest binding energy, an examination of the three-dimensional binding positions revealed that CNDs did not fit properly into the active site. In contrast, CNDs/*o*-PDA was found to exhibit a binding conformation similar to that of the reference ligand. This result indicates that evaluating the binding score alone may not be sufficient and that scores must be interpreted in conjunction with binding conformations. Indeed, in molecular docking analyses used to support findings on biological activity, it has been reported that various interactions—such as hydrogen bonds, van der Waals forces, and electrostatic interactions—must also be considered when evaluating the interactions of carbon quantum dots with biological targets [47]. Similarly, the stronger binding of MAVA/*o*-PDA to EGFR (−6.6 and −5.6 kcal/mol) compared to MAVA is consistent with MAVA/*o*-PDA exhibiting higher anticancer activity than MAVA in the experimental IC_50_ results. These results are consistent with the findings from our research group’s previous study. In that study, it was reported that functionalization with *o*-PDA increased the binding affinity of graphene-based conjugates to thymidylate synthase, and the lowest binding score was obtained for the *o*-PDA conjugate (−12.4 kcal/mol) [48]. When these findings are considered together, it is thought that functionalization with *o*-PDA may improve binding properties against different biological targets.

Based on the antimicrobial activity results, the high efficacy of CNDs/*o*-PDA and MAVA/*o*-PDA against Gram-negative bacteria compared to their pre-conjugation components—and even the fact that CNDs/*o*-PDA outperformed the standard antibiotic against *E. coli*—can be attributed to the mechanisms described for carbon quantum dot-based systems. In the literature, it has been reported that bacteria-affinity carbon dots exert a bactericidal effect by inhibiting an enzyme involved in bacterial cell wall synthesis [49], while *o*-phenylenediamine-derived carbon dots exert a bactericidal effect through the production of reactive oxygen species (ROS) [50]; these mechanisms offer a possible explanation for the efficacy of the conjugates in the present study. However, the fact that the conjugates were less effective against Gram-positive bacteria than the reference antibiotics can be explained by the finding reported in drug-functional polymer systems that the molecule’s level of hydrophobicity directly determines its efficacy against Gram-positive bacteria [10]; this suggests that the hydrophilic/hydrophobic balance of the conjugates may not be optimal for this group of bacteria.

Similarly, according to classification criteria widely used in the literature, MIC values are classified as effective (MIC ≤ 100 µg/mL), moderate (100 < MIC ≤ 625 µg/mL), and weak (MIC > 625 µg/mL) [44,45]. Based on this classification, it can be stated that both synthesized compounds (CNDs/*o*-PDA and MAVA/*o*-PDA) and their individual components exhibited effective MIC values against *E. faecalis* and *B. cereus*, and moderately effective MIC values against *S. mutans*. Similarly, the antifungal activity of the synthesized compounds against *C. albicans* was significantly lower than the MIC values of the reference antibiotics; however, this is due to the fact that the antifungal agent used as a reference itself has an extremely low MIC profile. Indeed, when evaluated according to general classification criteria in the literature, the synthesized compounds are seen to exhibit moderate antifungal activity against *C. albicans* [29,30].

Binding analyses targeting antimicrobial targets also showed a general trend similar to the experimental MIC results. For Gram-negative bacteria, binding energies ranged from −6.9 to −5.0 kcal/mol for *E. coli*, from −7.3 to −4.5 kcal/mol for *K. pneumoniae*, and from −6.7 to −4.9 kcal/mol for *P. aeruginosa*. In Gram-positive bacteria, these values ranged from −7.2 to −4.9 kcal/mol for *S. aureus*, from −7.7 to −5.0 kcal/mol for *E. faecalis*, from −7.3 to −4.4 kcal/mol for *S. mutans*, and from −7.3 to −4.6 kcal/mol for *B. cereus*. The values obtained for *C. albicans* (−8.0 to −5.2 kcal/mol), however, indicated a stronger binding tendency compared to the other targets. These results are consistent with previous studies reporting that carbon-based and polymer-based compounds can form moderate to strong interactions with various biological macromolecules. For example, it has been reported that nitrogen-doped carbon dots bind to human serum albumin with a binding score of −8.3 kcal/mol, and that hydrogen bonds and hydrophobic interactions play a role in this interaction [51]. From this perspective, the binding results obtained suggest that the antimicrobial effects of the conjugates under investigation may be supported by potential molecular interactions.

To establish a rational link between computational predictions and in vitro biological evaluations, the Epidermal Growth Factor Receptor (EGFR) was selected as a key molecular target. EGFR is a transmembrane tyrosine kinase that triggers tumor proliferation, survival, and progression, and is overexpressed in numerous malignancies. Analysis of cell line-based gene expression profiles from The Human Protein Atlas (HPA) database confirmed the functional presence of EGFR in the selected cancer models—including A549, MCF-7, MG-63, and C6 cells—thereby establishing a biologically meaningful framework for cytotoxicity assays. Consistent with experimental findings showing that *o*-PDA surface conjugation significantly lowered IC_50_ values (e.g., 6.50 µg/mL for MAVA/*o*-PDA in C6 cells and 9.42 µg/mL for CNDs/*o*-PDA in MG-63 cells) and improved selectivity indices, molecular docking studies performed against EGFR (PDB ID: 1XKK) provided mechanistic insights into target-ligand interactions. Notably, analysis of the 3D binding conformation revealed that while unfunctionalized CNDs failed to effectively position themselves within the catalytic pocket despite achieving a significant score, functionalization with *o*-PDA enabled an optimal conformational alignment similar to that of the co-crystallized ligand.

The results of this study indicate that molecular docking analyses may be useful not only for evaluating target-ligand interactions but also for investigating potential molecular interactions between carrier structures and active agents. In the literature, it has been reported that the interaction and binding affinity of doxorubicin with transferrin-functionalized carbon quantum dots were evaluated using molecular docking methods, and that this approach could contribute to the development of targeted drug delivery systems [46]. Similarly, in a PEGylated graphene oxide quantum dot–chitosan-based nanotheranostic system, the interaction between the MUC-1 aptamer and its receptor, as well as the potential effects of the ZnO/chitosan quantum dot nanocomposite on the donepezil–albumin interaction, were investigated using docking analyses [52,53]. When considered together with these studies, the docking results obtained for CNDs and MAVA in the present study suggest that these structures are candidate carrier systems that could be used to investigate their interactions with various biological targets. However, the current results do not directly indicate drug-carrying capacity; they suggest that the potential of CNDs and MAVA to serve as carriers in conjugate systems formed with active agents other than *o*-PDA—such as chemotherapeutic drugs—should be evaluated in future studies.

The molecular weight, TPSA value, number of rotatable bonds, and number of hydrogen bond donors/acceptors of CNDs/*o*-PDA have increased significantly compared to CNDs; this has caused the molecule to violate the three Lipinski rules (MW > 500, HBA > 10, HBD > 5) and, consequently, to have a low bioavailability score (0.11). In contrast, MAVA-*o*-PDA maintained moderate bioavailability potential (0.56) without violating any of the Lipinski rules. This finding highlights a significant difference between the two conjugates: while the strong biological activity exhibited by MAVA-*o*-PDA aligns with a suitable pharmacokinetic profile, the similarly strong activity of CNDs/*o*-PDA is accompanied by a weaker theoretical bioavailability profile. This suggests that the high efficacy demonstrated by CNDs/o-PDA under in vitro conditions may not be fully maintained in systemic applications, and therefore it may be beneficial to evaluate this conjugate in terms of alternative routes of administration or appropriate formulation strategies.

In terms of gastrointestinal absorption and blood–brain barrier (BBB) permeability, it is noteworthy that *o*-PDA is the only molecule capable of crossing the BBB; CNDs, CNDs/*o*-PDA, and MAVA/*o*-PDA are unable to cross this barrier. This finding suggests that the conjugates may exert a targeted effect in peripheral tissues without posing a risk of central nervous system toxicity, which can be considered a positive safety feature, particularly in anticancer applications.

Toxicity predictions further support this picture. Analysis of the ProTox 3.0 prediction results revealed that CNDs and CND/*o*-PDA compounds were classified as toxicity class 6, MAVA and *o*-PDA as toxicity class 4, and MAVA/*o*-PDA as toxicity class 5. However, it should be noted that while such in silico toxicity classifications provide predictions about the toxicological properties of compounds, they may not, on their own, fully reflect all aspects of biological effects. In particular, classifications based on different toxicological parameters must be addressed with a careful and comprehensive approach when evaluating the overall toxicity profile of compounds. Therefore, ProTox results should be interpreted with caution as a predictive assessment of the toxicological properties of compounds.

While the in silico findings of this study provide a valuable framework for interpreting the experimental results, they are based on computational predictions and therefore need to be validated by in vitro and in vivo studies. Furthermore, it should be noted as a limitation that, because molecular docking analysis provides a static model, it may not fully reflect the behavior of target protein-ligand interactions under actual conditions in biological systems.

The use of a broad panel of microorganisms—including four different cancer cell lines, one healthy cell line, and various Gram-negative and Gram-positive bacteria as well as a fungal species—strengthens the scope of the findings. However, the molecular mechanisms underlying the anticancer and antimicrobial effects were not directly investigated in this study.

When evaluating these findings, certain limitations of the study should be taken into account. Despite the increase in anticancer and antimicrobial activities observed following conjugation with *o*-PDA, the underlying molecular mechanisms of these effects have not been experimentally validated. Future studies that utilize different normal and cancer cell models, experimentally investigate the relevant molecular pathways, and validate efficacy, selectivity, pharmacokinetic properties, and safety profiles through in vivo studies will contribute to a more comprehensive understanding of the biological advantages provided by *o*-PDA conjugation.

Overall, the findings of this study demonstrate that the conjugation of CNDs and MAVA with *o*-PDA consistently enhances anticancer and antimicrobial activity, although these two conjugates exhibit different physicochemical and toxicological profiles. While MAVA/*o*-PDA stands out as a more balanced candidate, offering both strong biological activity and acceptable pharmacokinetic properties, the superior in vitro activity of CNDs/*o*-PDA may require support through formulation strategies due to its limited theoretical bioavailability. Furthermore, with regard to the comparison of flat and spherical carrier structures for *o*-PDA—one of the study’s primary objectives—the findings suggest that the flat-chain polymer backbone, represented by MAVA, may be a more suitable carrier structure for the binding of *o*-PDA.

## 5. Conclusions

In this study, the effects of *o*-PDA conjugation with CNDs and MAVA—two carrier structures with the same surface functionality—on anticancer and antimicrobial activities were evaluated comparatively. The findings indicated that *o*-PDA conjugation generally enhanced biological activity for both CNDs and MAVA. Molecular docking analyses revealed potential interactions that support the experimental findings, while in silico pharmacokinetic and toxicity assessments showed that the two conjugates have different profiles. Overall, while MAVA/*o*-PDA emerged as a more advantageous candidate due to its strong biological activity and more balanced predicted pharmacokinetic properties, CNDs/*o*-PDA exhibited lower theoretical bioavailability despite its high in vitro activity. These results suggest that the type of carrier and the conjugate structure may influence the biological properties of the pharmaceutically active compounds and that MAVA may be a suitable carrier structure for *o*-PDA. However, these findings need to be validated through mechanistic and in vivo studies.

## Figures and Tables

**Table 1:** IC_50_ values for CNDs, *o*-PDA, CNDs/*o*-PDA, MAVA, and MAVA/*o*-PDA.

Test Materials	C6	MCF-7	MG-63	A549	WI-38
*CNDs, o-PDA and CNDs/o-PDA*					
CNDs	19.55 ± 0.25	17.61 ± 0.17	22.29 ± 0.45	17.02 ± 0.17	>100
*o*-PDA	20.53 ± 0.14	19.07 ± 0.13	16.82 ± 0.63	14.69 ± 0.13	>100
CNDs/*o*-PDA	11.47 ± 0.35	13.28 ± 0.16	9.42 ± 0.54	12.06 ± 0.36	>100
*p*^1^-value	*p* < 0.00001	*p* < 0.00001	*p* < 0.00001	*p* < 0.00001	1.00
*MAVA, o-PDA and MAVA/o-PDA*					
*o*-PDA	20.53 ± 0.14	19.07 ± 0.13	16.82 ± 0.63	14.69 ± 0.13	>100
MAVA	12.88 ± 0.96	19.94 ± 0.17	19.66 ± 0.15	16.15 ± 0.24	>100
MAVA/*o*-PDA	6.50 ± 0.01	14.81 ± 0.09	15.05 ± 0.28	15.28 ± 0.06	>100
*p*^2^-value	*p* < 0.00001	*p* < 0.00001	*p* < 0.00001	*p* < 0.00001	1.00
*CNDs* vs. *MAVA*					
CNDs	19.55 ± 0.25	17.61 ± 0.17	22.29 ± 0.45	17.02 ± 0.17	>100
MAVA	12.88 ± 0.96	19.94 ± 0.17	19.66 ± 0.15	16.15 ± 0.24	>100
*p*^3^-value	0.0009	0.9	0.005	0.34	1.00
*CNDs/o-PDA* vs. *MAVA/o-PDA*					
CNDs/*o*-PDA	11.47 ± 0.35	13.28 ± 0.16	9.42 ± 0.54	12.06 ± 0.36	>100
MAVA/*o*-PDA	6.50 ± 0.01	14.81 ± 0.09	15.05 ± 0.28	15.28 ± 0.06	>100
*p*^4^-value	*p* < 0.0001	0.12	0.08	0.00003	1.00
*Doxorubicin*	1.82 ± 0.13	2.58 ± 0.29	2.35 ± 0.21	1.27 ± 0.11	9.54 ± 1.65

Normality of data distribution was assessed using the Shapiro–Wilk test. Comparisons between two groups were performed using the independent-samples t-test for normally distributed data or the Mann–Whitney U test for non-normally distributed data. Comparisons among three or more groups were performed using one-way ANOVA followed by Tukey’s HSD post hoc test for normally distributed data, or the Kruskal–Wallis test followed by Dunn’s post hoc test for non-normally distributed data. *p*^1^ represents the comparison among the CNDs, *o*-PDA, and CNDs/*o*-PDA groups; *p*^2^ represents the comparison among the *o*-PDA, MAVA, and MAVA/*o*-PDA groups; *p*^3^ represents the comparison between the CNDs and MAVA groups; and *p*^4^ represents the comparison between the CNDs/*o*-PDA and MAVA/*o*-PDA groups. All comparisons were performed separately for each cell line (C6, MCF-7, MG-63, A-549, and WI-38). Data are presented as mean ± SD. A *p*-value < 0.05 was considered statistically significant.

**Table 2:** Selectivity Index (SI) values for CNDs, *o*-PDA, CNDs/*o*-PDA, MAVA, and MAVA/*o*-PDA.

Test Materials		Selectivity Index (SI)		
	C6	MCF-7	MG-63	A549
CNDs	5.12	5.68	4.49	5.88
*o*-PDA	4.87	5.24	5.95	6.81
CNDs/*o*-PDA	8.72	7.53	10.62	8.29
MAVA	9.19	5.02	5.09	6.19
MAVA/*o*-PDA	15.38	6.75	6.67	6.55
Doxorubicin	5.24	3.69	4.06	7.51

**Table 3:** MIC results for CNDs, *o*-PDA, CNDs/*o*-PDA, MAVA, and MAVA/*o*-PDA (µg/mL).

Bacteria and Yeast	MIC (µg/mL) CNDs	MIC (µg/mL) *o*-PDA	MIC (µg/mL) CNDs/*o*-PDA	MIC (µg/mL) MAVA	MIC (µg/mL) MAVA/*o*-PDA	MIC (µg/mL) Antibiotics	Antibiotics
*Escherichia coli*	100	100	25	>200	50	32	Amoxicillin/Clavulanic acid
*Klebsiella pneumoniae*	>200	>200	100	>200	100	32	Amoxicillin/Clavulanic acid
*Pseudomonas aeruginosa*	>200	100	25	>200	25	16	Amikacin
*Staphylococcus aureus*	100	100	50	>200	50	16	Amikacin
*Enterococcus faecalis*	100	>200	50	>200	50	8	Amoxicillin
*Streptococcus mutans*	>200	>200	100	>200	100	2	Amoxicillin
*Bacillus cereus*	100	>200	50	>200	50	2	Vancomycin
*Candida albicans*	>200	100	100	>200	100	0.25	Fluconazole

MIC: Minimum Inhibitory Concentration.

**Table 4:** Summary of the anticancer target tested.

TARGET	CNDs	*o*-PDA	CNDs/*o*-PDA	MAVA	MAVA/*o*-PDA	Co-Crystal
EGFR	−7.9	−5.1	−5.3	−5.6	−6.6	−11.6

**Table 5:** Evaluation of antimicrobial activity against Gram-positive bacteria, Gram-negative bacteria, and yeast *Candida albicans*.

Target	CNDs	*o*-PDA	CNDs/*o*-PDA	MAVA	MAVA/*o*-PDA	Cocrystal
*Escherichia coli*	−6.9	−5	−6.8	−5.8	−6.3	−8.7
*Klebsiella pneumoniae*	−7.3	−4.5	−7.3	−5	−6.8	−5.8
*Pseudomonas aeruginosa*	−6.7	−4.9	−5.7	−5.2	−6.5	−7.5
*Staphylococcus aureus*	−7.2	−4.9	−6.7	−6	−6.5	−8.8
*Enterococcus faecalis*	−7.7	−5.0	−5.5	−5.2	−6.8	−8.6
*Streptococcus mutans*	−7.3	−4.4	−5.6	−4.8	−5.5	−6.2
*Bacillus cereus*	−7.3	−4.6	−5.7	−4.9	−6.2	−7.2
*Candida albicans*	−7.7	−5.2	−6.8	−5.9	−8	−12.1

**Table 6:** In silico physicochemical properties of the tested compounds.

Test Materials	MW	Rotatable Bonds	H-Bond Acceptors	H-Bond Donors	TPSA	WLOGP	Consensus Log P
CNDs	400.29	0	12	0	173.48	−0.6	−0.35
CNDS-*o*-PDA	824.79	16	12	12	369.68	1.42	−0.78
MAVA	212.2	4	5	0	69.67	0.73	1.14
MAVA-*o*-PDA	320.34	8	5	3	118.72	1.77	1.38
*o*-PDA	108.14	0	0	2	52.04	0.87	0.61

**Table 7:** In silico pharmacokinetic properties and drug-likeness of the tested compounds.

Test Materials	GI Absorption	BBB Permeant	Pgp Substrate	Log Kp (cm/s)	Lipinski #Violations	Bioavailability Score
CNDs	Low	No	No	−10.06	1	0.55
CNDS-*o*-PDA	Low	No	Yes	−12.2	3	0.11
MAVA	High	No	No	−7	0	0.55
MAVA-*o*-PDA	High	No	No	−7.44	0	0.56
*o*-PDA	High	Yes	No	−6.85	0	0.55

## Data Availability

The original contributions presented in this study are included in the article and Supplementary Materials. Further inquiries can be directed to the corresponding author.

## Supplementary Materials

The following supporting information can be downloaded at: https://www.mdpi.com/article/10.3390/biom16101478/s1. Figure S1: EGFR expression profiles in various cancer types and cell lines based on The Human Protein Atlas data. Figure S2: Visualization of the binding sites of CNDs, CNDs-*o*-PDA, MAVA, MAVA-*o*-PDA, and *o*-PDA for binding-region analysis against *Staphylococcus aureus*. Figure S3: Visualization of the binding sites of CNDs, CNDs-*o*-PDA, MAVA, MAVA-*o*-PDA, and *o*-PDA for binding-region analysis against *Enterococcus faecalis*, Figure S4. Visualization of the binding sites of CNDs, CNDs-*o*-PDA, MAVA, MAVA-*o*-PDA, and *o*-PDA for binding-region analysis against *Streptococcus mutans*. Figure S5. Visualization of the binding sites of CNDs, CNDs-*o*-PDA, MAVA, MAVA-*o*-PDA, and *o*-PDA for binding-region analysis against *Pseudomonas aeruginosa*. Figure S6. Visualization of the binding sites of CNDs, CNDs-*o*-PDA, MAVA, MAVA-*o*-PDA, and *o*-PDA for binding-region analysis against *Escherichia coli*. Figure S7. Visualization of the binding sites of CNDs, CNDs-*o*-PDA, MAVA, MAVA-*o*-PDA, and *o*-PDA for binding-region analysis against *Klebsiella pneumoniae*. Figure S8. Visualization of the binding sites of CNDs, CNDs-*o*-PDA, MAVA, MAVA-*o*-PDA, and *o*-PDA for binding-region analysis against *Bacillus cereus*. Figure S9. Visualization of the binding sites of CNDs, CNDs-*o*-PDA, MAVA, MAVA-*o*-PDA, and *o*-PDA for binding-region analysis against *Candida albicans*. Table S1. Color legend for the different types of molecular interactions in Discovery Studio Visualizer.
