# Supplementary material for: Comparative In Vitro and In Silico Biological Evaluation of o-Phenylenediamine Conjugates Based on Isofunctional-Surfaced Polyanhydride and Citric Acid-Derived Carbon Quantum Nanodots
Source: Biomolecules. 2026 Oct 11;16(10):1478. doi: 10.3390/biom16101478 (PMC13702659; doi:10.3390/biom16101478)
Supplement: Supplementary file 1 [file biomolecules-16-01478-s001.zip › biomolecules-4586715-supplementary.pdf]

Supplementary File

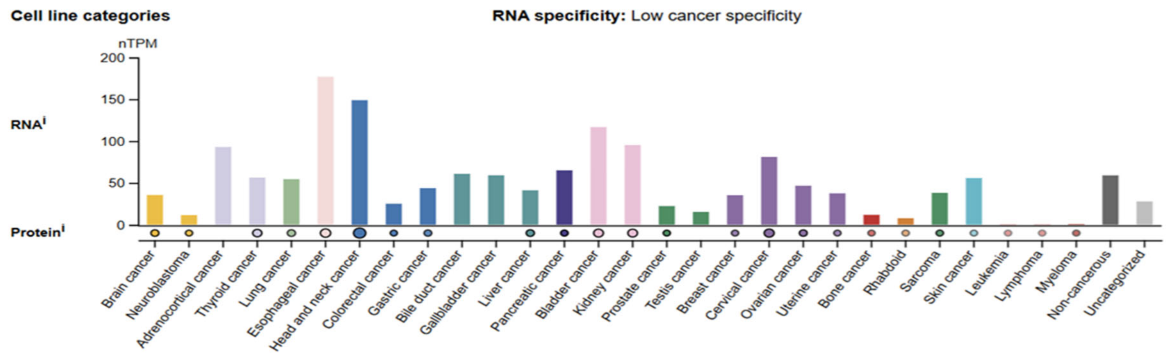

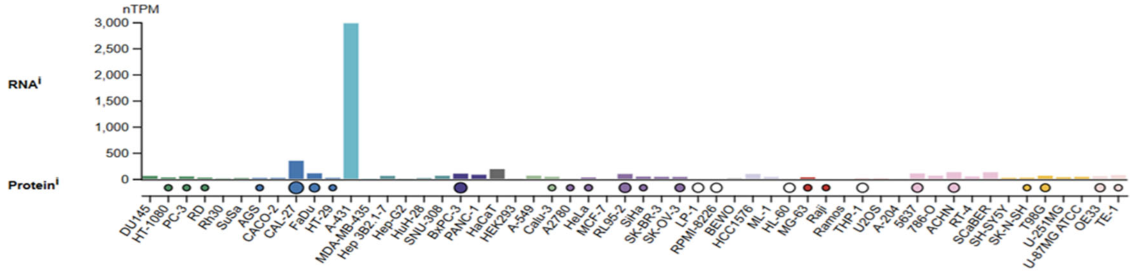

Bone cancer (n=21)

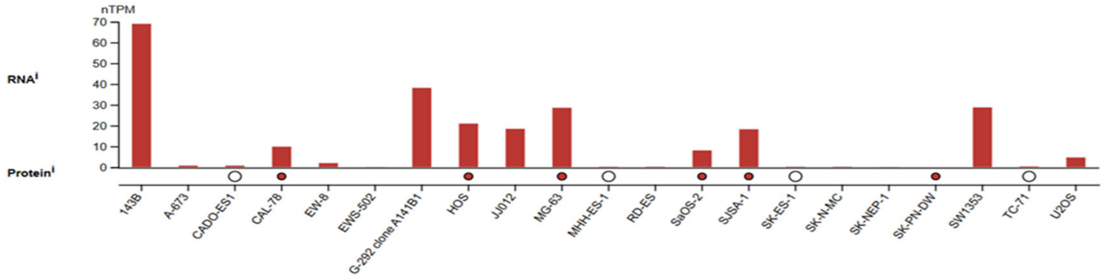

Breast cancer (n=62)

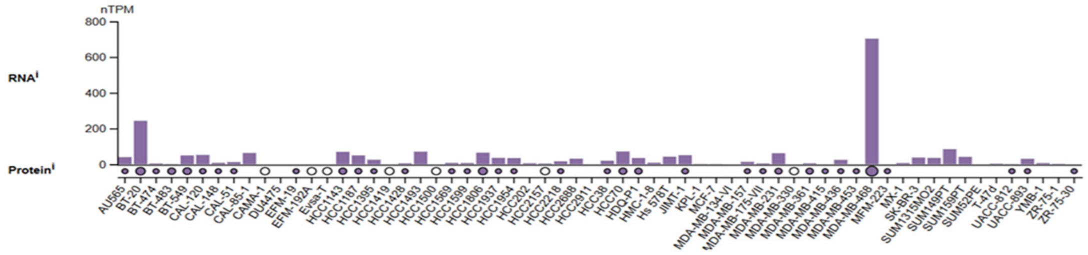

Lung cancer (n=232)

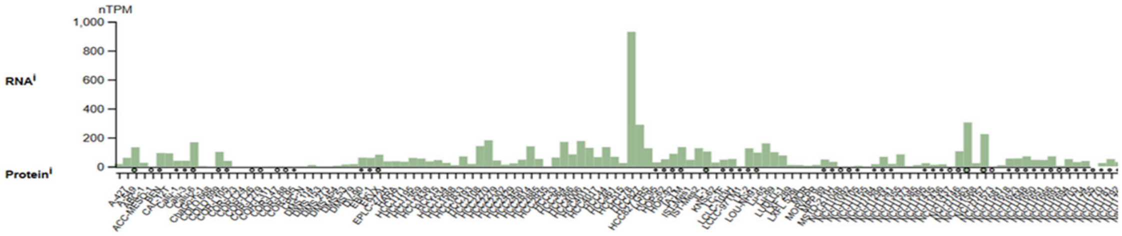

Brain cancer (n=80)

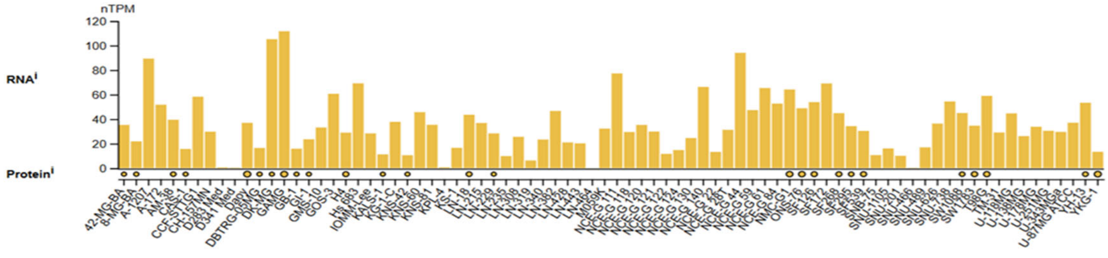

Figure S1. EGFR expression profiles in various cancer types and cell lines based on The Human Protein Atlas data.

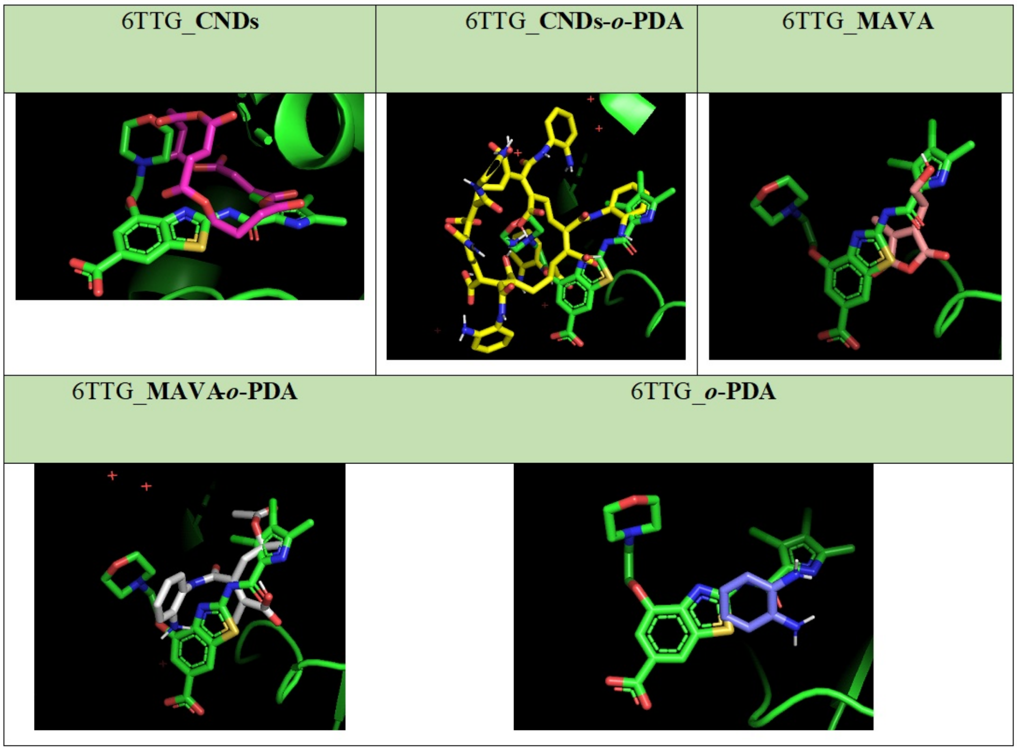

Figure S2. Visualization of the binding sites of CNDs, CNDs-o-PDA, MAVA, MAVA-o-PDA, and o-PDA for binding-region analysis against *Staphylococcus aureus*.

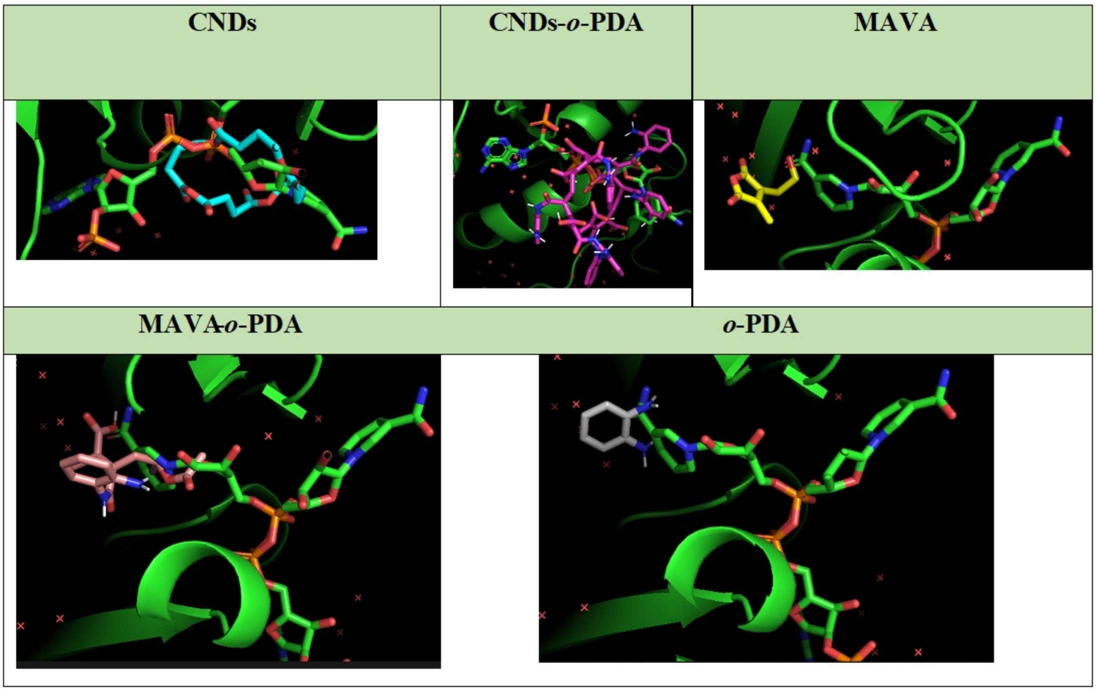

Figure S3. Visualization of the binding sites of CNDs, CNDs-o-PDA, MAVA, MAVA-o-PDA, and o-PDA for binding-region analysis against *Enterococcus faecalis*.

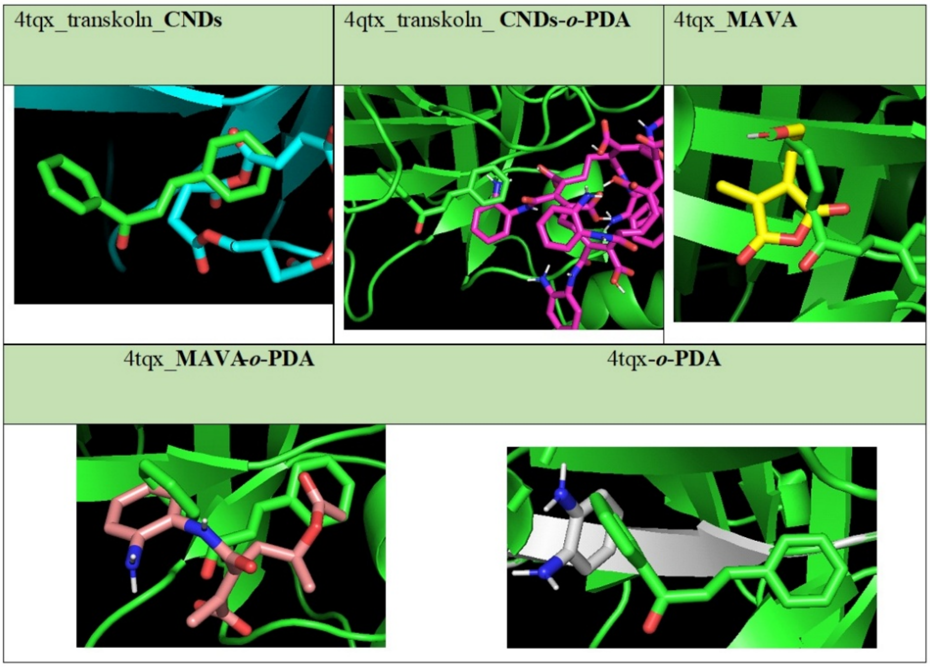

Figure S4. Visualization of the binding sites of CNDs, CNDs-o-PDA, MAVA, MAVA-o-PDA, and o-PDA for binding-region analysis against *Streptococcus mutans.*

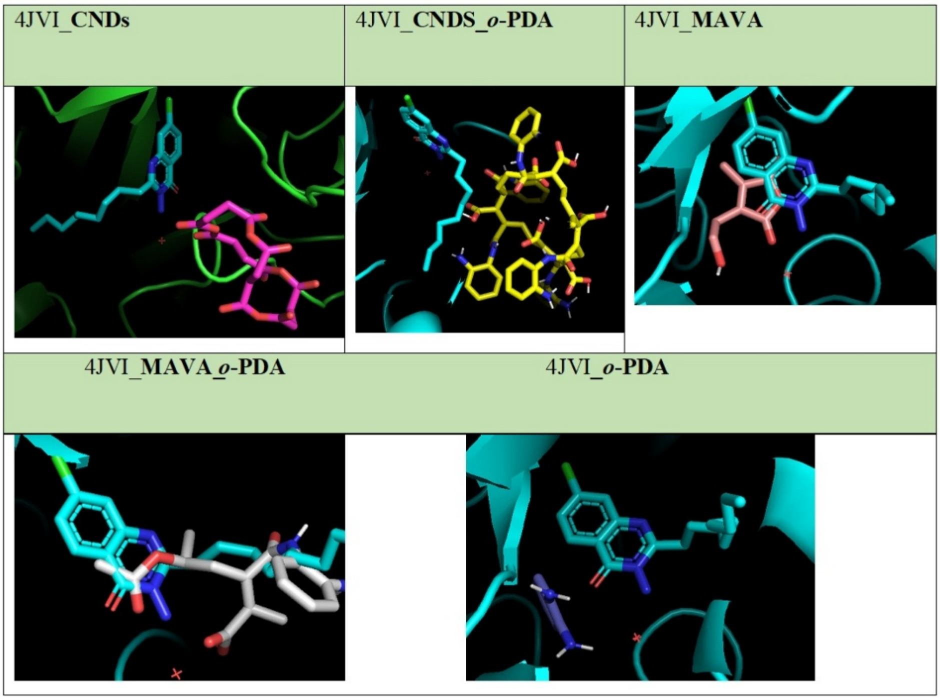

Figure S5. Visualization of the binding sites of CNDs, CNDs-o-PDA, MAVA, MAVA-o-PDA, and o-PDA for binding-region analysis against *Pseudomonas aeruginosa.*

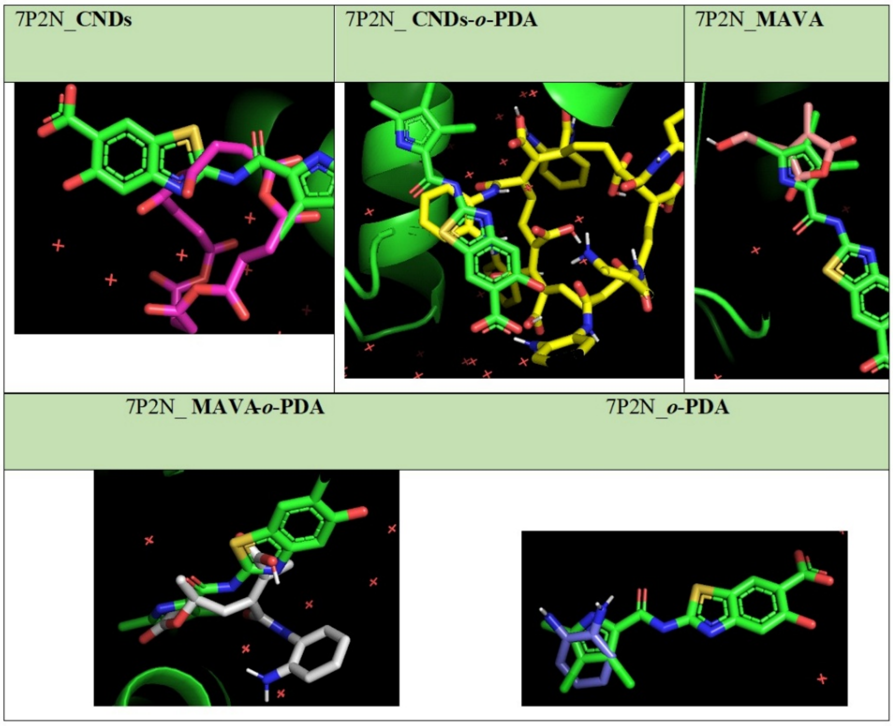

Figure S6. Visualization of the binding sites of CNDs, CNDs-o-PDA, MAVA, MAVA-o-PDA, and o-PDA for binding-region analysis against *Escherichia coli.*

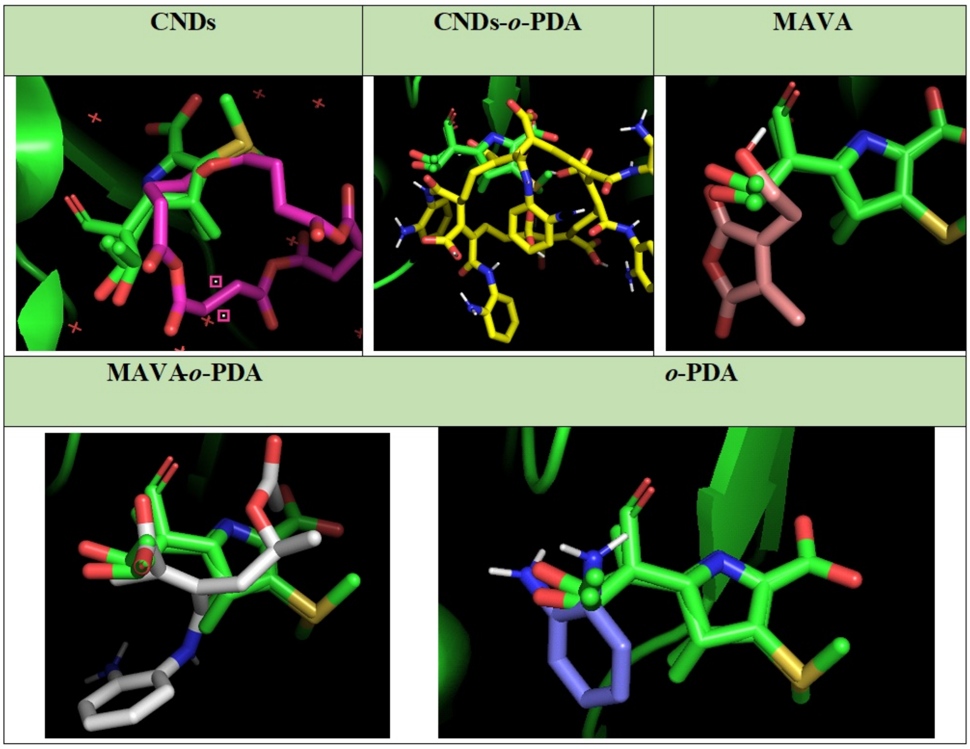

Figure S7. Visualization of the binding sites of CNDs, CNDs-o-PDA, MAVA, MAVA-o-PDA, and o-PDA for binding-region analysis against *Klebsiella pneumoniae.*

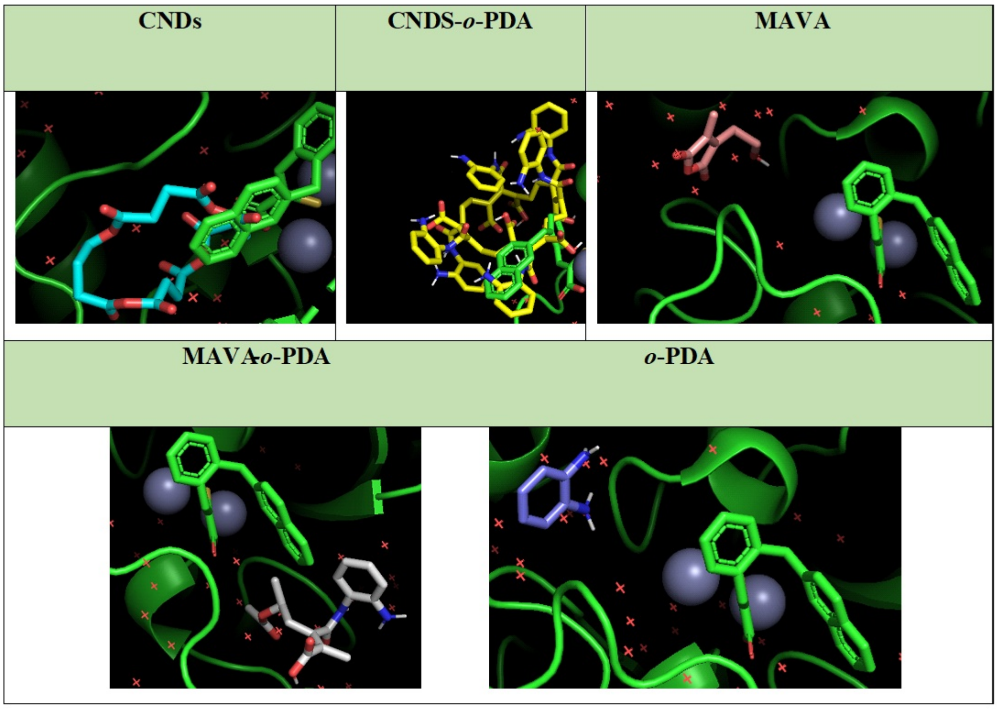

Figure S8. Visualization of the binding sites of CNDs, CNDs-o-PDA, MAVA, MAVA-o-PDA, and o-PDA for binding-region analysis against *Bacillus cereus.*

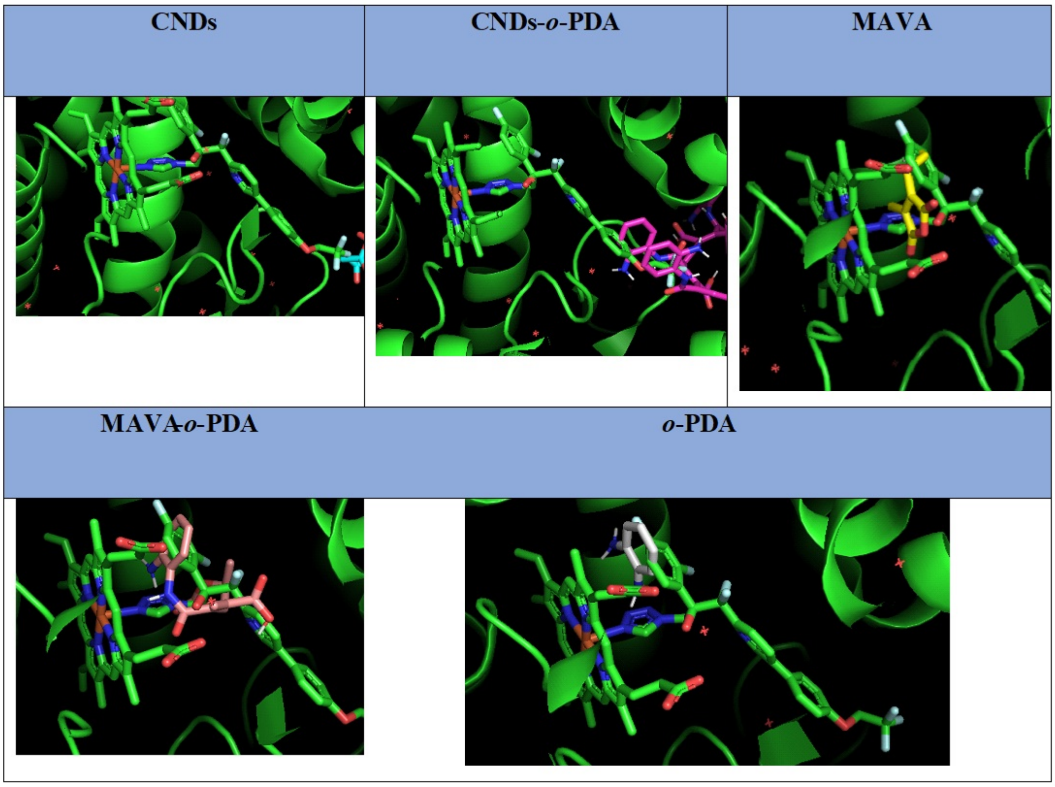

Figure S9. Visualization of the binding sites of CNDs, CNDs-o-PDA, MAVA, MAVA-o-PDA, and o-PDA for binding-region analysis against *Candida albicans.*

Table S1. Color legend for the different types of molecular interactions in Discovery Studio Visualizer

van der Waals	Conventional Hydrogen Bond	Carbon Hydrogen Bond
Pi-Donor Hydrogen Bond	Unfavorable Negative-Negative	Unfavorable Donor-Donor
Unfavorable Acceptor-Acceptor	Pi-Anion	Attractive Charge
Pi-Sigma	Pi-Alkyl	Amide-Pi Stacked
Alkyl	Pi-Pi Stacked	Pi-Pi T-shaped
Salt Bridge	Pi-Sulfur	
